# How Do Liquid-Junction Potentials and Medium Polarity at Electrode Surfaces Affect Electrochemical Analyses for Charge-Transfer Systems?

**DOI:** 10.1021/acs.jpcb.2c07983

**Published:** 2023-02-03

**Authors:** Maximillian F. Mayther, Omar O’Mari, Paul Flacke, Dev Bhatt, Samantha Andrews, Valentine I. Vullev

**Affiliations:** † Department of Chemistry, University of California, Riverside, California92521, United States; ⊥ Department of Bioengineering, University of California, Riverside, California92521, United States; § Department of Biochemistry, University of California, Riverside, California92521, United States; ∥ Materials Science and Engineering Program, University of California, Riverside, California92521, United States

## Abstract

The importance of electrochemical analysis for charge-transfer science cannot be overstated. Interfaces in electrochemical cells present certain challenges in the interpretation and the utility of the analysis. This publication focuses on: (1) the medium polarity that redox species experience at the electrode surfaces that is smaller than the polarity in the bulk media and (2) the liquid-junction potentials from interfacing electrolyte solutions of different organic solvents, namely, dichloromethane, benzonitrile, and acetonitrile. Electron-donor–acceptor pairs of aromatics with similar structures (i.e., 1-naphthylamine and 1-nitronaphthalene, 10-methylphenothiazine and 9-nitroanthracene, and 1-aminopyrene and 1-nitropyrene) serve as redox analytes for this study. Using the difference between the reduction potentials of the oxidized donors and the acceptors eliminates the effects of the liquid junctions on the analysis of charge-transfer thermodynamics. This analysis also offers a means for evaluating the medium polarity that the redox species experience at the surface of the working electrode and the effects of the liquid junctions on the measured reduction potentials. While the liquid-junction potentials between the dichloromethane and acetonitrile solutions amount to about 90 mV, for the benzonitrile-acetonitrile junctions, the potentials are only about 30 mV. The presented methods for analyzing the measured electrochemical characteristics of donors and acceptors illustrate a means for improved evaluation of the thermodynamics of charge-transfer systems.

## Introduction

Charge transfer (CT), in its many forms, is fundamental for sustaining not only life on Earth, but also our modern ways of living.^1^ Electrochemical analysis is crucial for characterizing the thermodynamics of CT and photoinduced charge transfer (PCT).^2^ Specifically, the reduction potentials, *E*^(0)^, of an acceptor and the oxidized form of a donor are essential for estimating the driving forces of ground-state electron transfer (ET) and the PCT between them:^1−4^
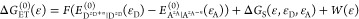
1a
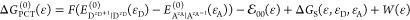
1bIn eq 1, *F* is the Faraday constant;  is the zero-to-zero optical excitation energy (1) of the donor for photoinduced electron transfer (PET), and (2) of the acceptor for photoinduced hole transfer (PHT); *z*_A_ and *z*_D_ are the initial charges of the acceptor and the donor, respectively; and *n* is the number of transferred electrons during ET. Relating optical and electrochemical characteristics obtained from samples with different static dielectric constants is a principal feature of eq 1. Specifically, ε is the static dielectric constant of the medium used for the ET and PCT, and for recording the spectra needed for estimating , while ε_A_ and ε_D_ are the dielectric constants of the solutions employed for measuring the reduction potentials of the acceptor and the oxidized donor, respectively. The Born solvation term, Δ*G*_S_, accounts for interactions of the donor and the acceptor with the solvating media and corrects the reduction potentials from their values for ε_D_ and ε_A_ to values for ε.^5^ The Coulomb work term, *W*, accounts for the difference in the donor–acceptor electrostatic interactions before and after the CT step.^3^

The broad use of eq 1 for characterizing CT processes in biology, chemistry, physics, and engineering testifies to its importance.^1^ The scientific community, in general, refers to eq 1b as the Rehm–Weller equation. As defined by IUPAC, however, the Rehm–Weller equation represents the empirical correlation between the second-order rate constants and the driving force of bimolecular CT that does not exhibit Marcus-like behavior.^6^ Nevertheless, calling eq 1b the “Rehm–Weller equation” reflects acknowledging Weller and Rehm for their introduction of the Coulomb term, *W*, to the sum of potentials and  for calculating PCT driving forces.^3^

Heterogenous electrochemical oxidation and reduction provide the experimental means for estimating the reduction potentials needed for evaluating the thermodynamics of not only heterogeneous, but also homogeneous, ET and PCT. How does one evaluate ε_A_ and ε_D_ at the surface of the working electrode where the heterogeneous ET occurs? The electrolyte, added to the analyte solutions for ensuring sufficient electrical conductivity, introduces challenges for estimating the polarity of the microenvironment where the electrochemical reduction and oxidation occur. For example, the Gouy–Chapman–Stern (GCS) model offers a means for estimating the increased concentration of ions in the Helmholtz and the diffused layers at the surfaces of polarized electrodes.^7−9^ The GCS formalism, however, assumes a static dielectric constant, ε, at the electrode surfaces that is identical to ε of the bulk solution, and often approximated to the dielectric constant of the solvent. Conversely, an increase in electrolyte concentration (*C*_*el*_) can substantially increase or decrease the dielectric constant of a solution, depending on if the ions have chaotropic or kosmotropic effects on the solvent structure.^10−14^ Furthermore, the dielectric properties of the double layers at electrode surfaces, where the electrochemical reduction and oxidation occur, differ from the dielectric properties of the bulk electrolyte solution. Impeding the molecular and ionic motions in the Helmholtz and the diffused layers, under applied potential, decreases the orientational polarization in the microenvironment at the electrode surface. Such *electrofreezing* can substantially decrease the dielectric constant of the double layer.^15,16^ Dependence of the measured reduction potentials on *C*_*el*_ provides patterns that allow extrapolating potential values to *C*_*el*_ = 0 M, corresponding to the dielectric constants of the neat solvents.^17^ Nevertheless, these dielectric constants represent the properties of the solvents at the electrode surface.

Liquid junctions, interfacing two different solutions, usually via porous media, present another key challenge for electrochemical analysis.^18−22^ Differences in the transport properties and the activities of the ions on the two sides of a junction induce a sizable potential. In electrochemical cells, such liquid-junction potentials (*E*_LJ_) formed along the electrical paths between the working and the reference electrodes add to the measured voltages. Using solutions of miscible solvents and electrolytes composed of ions with similar mobility through the junctions provides a means for minimizing *E*_LJ_. Employing cell setups with pseudoreference electrodes that do not contain liquid junctions eliminates *E*_LJ_ altogether. The potentials of pseudoreference electrodes, however, depend on the compositions of the analyte solutions in which they are immersed, warranting caution in the interpretation of the measurements.

Herein, investigation of the electrochemical properties of pairs of electron donors and electron acceptors not only provides insight into the decreased medium polarity that the redox species experience at the surface of the working electrode, but also allows estimating *E*_LJ_ between different organic solutions comprising the same electrolyte. The results set a key perspective about the implementation of electrochemical data for evaluation of CT thermodynamics (eq 1).

## Results

### Electron Donors and Acceptors

**Figure 1:**
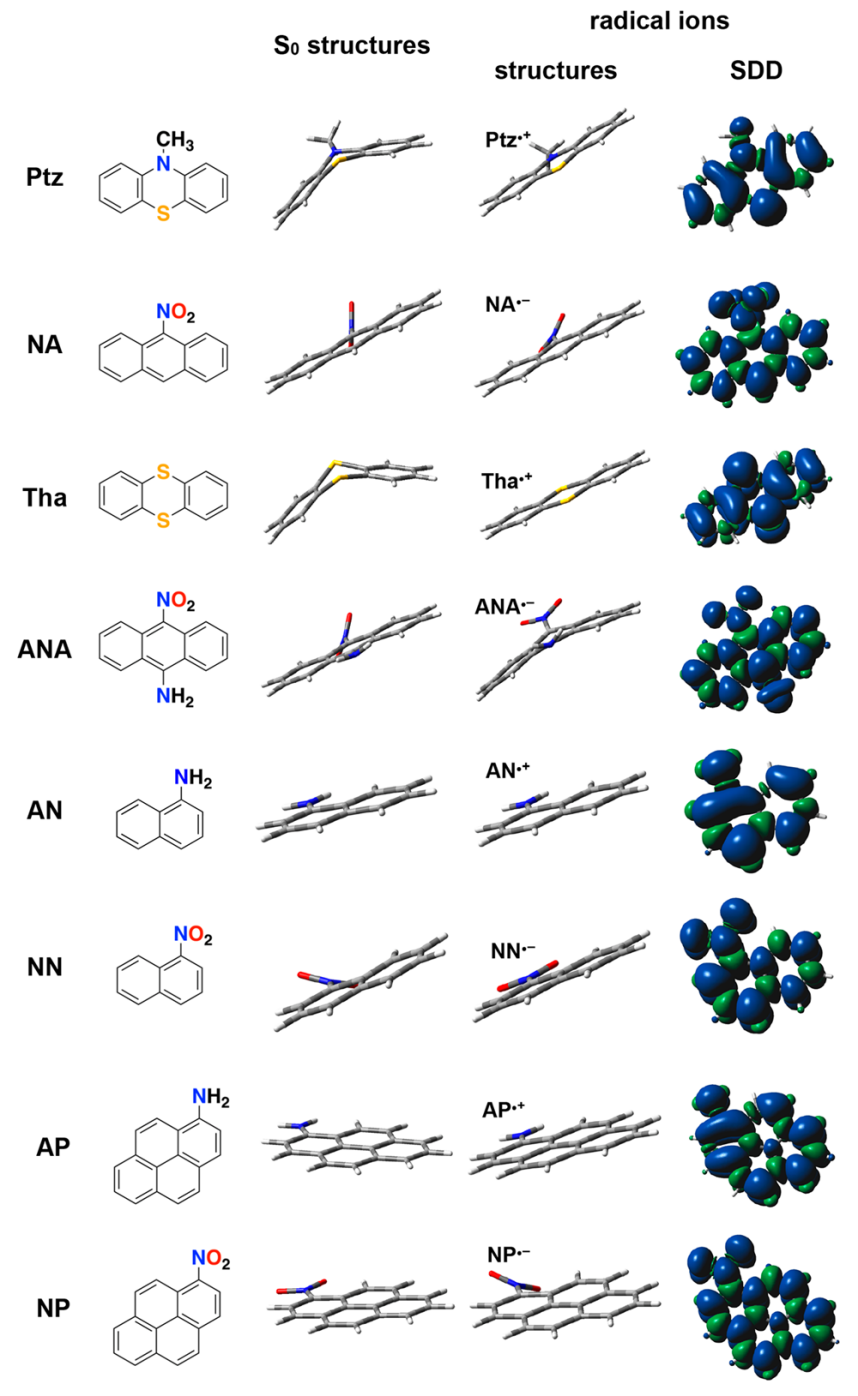
Structures of 10-methylphenothiazine (Ptz), 9-nitroanthracene (NA), thianthrene (Tha), 9-amino-10-nitroanthracene (ANA), 1-naphthylamine (AN), 1-nitronaphthalene (NN), 1-aminopyrene (AP), and 1-nitropyrene (NP) along with the structures and the electron spin-density distribution (SDD) of their radical ions, obtained from DFT calculations at the B3LYP/6-311+g(d,p)-gd3 level of theory with acetonitrile solvation media as implemented by the polarizable-continuum model (PCM). Blue represents the excess density of spin up, and green, of spin down.

For this study, we focus on pairs of electron donors and acceptors not only with similar structures, but also with similar spin-density distributions of their radical ions. The electron-rich 1-aminonaphthalene (AN) and the electron-deficient 1-nitronaphthalene (NN) have the same ring structure and a single substituent at the same position. The same is valid for the pairs of 10-methylphenothiazine (Ptz) and 9-nitroanthracene (NA), and of 1-aminopyrene (AP) and 1-nitropyrene (NP) (Figure 1). In addition to the similar sizes, the radical ions of the species of each of these donor–acceptor pairs, AN^•+^ and NN^•–^, Ptz^•+^ and NA^•–^, and AP^•+^ and NP^•–^, have similar spin-density distributions that spread over all of their aromatic rings (Figure 1).

Except for Ptz, all of these polycyclic aromatics and their radical ions have planar structures. In its ground state, Ptz assumes a bent structure, while its singly oxidized form, Ptz^•+^, is planar (Figure 1). This oxidation-induced conformational change is not unusual for such sulfur heterocyclic compounds. Thianthrene (Tha), for example, containing sulfurs at the two middle positions, exhibits the same oxidation-induced flattening of its bent ground-state structure (Figure 1).^23−25^ Furthermore, this type of behavior extends beyond heterocyclic structures. For instance, the planar ground-state structure of 9-amino-10-nitroanthracene (ANA) bends at carbons 9 and 10 upon single-electron reduction (Figure 1). These oxidation- and reduction-induced conformational changes present an attractive paradigm for molecular optically activatable mechanotransducers.

As UV absorbers, phenothiazine and many of its derivatives serve as electron donors for a range of CT systems.^26−28^ Conversely, certain phenothiazine derivatives, such as promethazine, chlorpromazine, and methotrimeprazine, exhibit biological activity as H_1_ blockers, i.e., antihistamines, and have a clinical significance as antiemetic and antipsychotic medications.^29−35^ The antiviral properties of some of them raised quite an interest during the COVID-19 pandemic as potential agents against the SARS-CoV-2 virus.^36−38^

The optical absorption of NA and NP extends into the visible spectral region. The immensely short lifetimes of the singlet excited sates of the vast majority of nitroaromatics, however, render them unfeasible for photosensitizers, unless they photoinitiated (sub)picosecond processes.^39−42^ With the femtosecond and picosecond lifetimes of their singlet excited states, NN, NA, and NP are not an exception.^41−43^ While adding electron-donating substituents to NN can substantially increase the lifetime of its singlet excited state,^42^ NN itself manifests subpicosecond intersystem crossing that is among the fastest for organic compounds. Therefore, AN and NN, Ptz and NA, and AP and NP can be good couples for photoinduced charge separation (CS) only when the donor–acceptor electronic coupling is sufficiently strong, and the reorganization energy matches the CT driving forces to ensure ultrafast femtosecond CS rates. Nevertheless, the structural similarities between AN and NN, Ptz and NA, and AP and NP make them good donor–acceptor pairs for testing the herein-presented electrochemical analysis for CT systems.

### Effects of Solvating Media on Reduction Potentials

**Figure 2:**
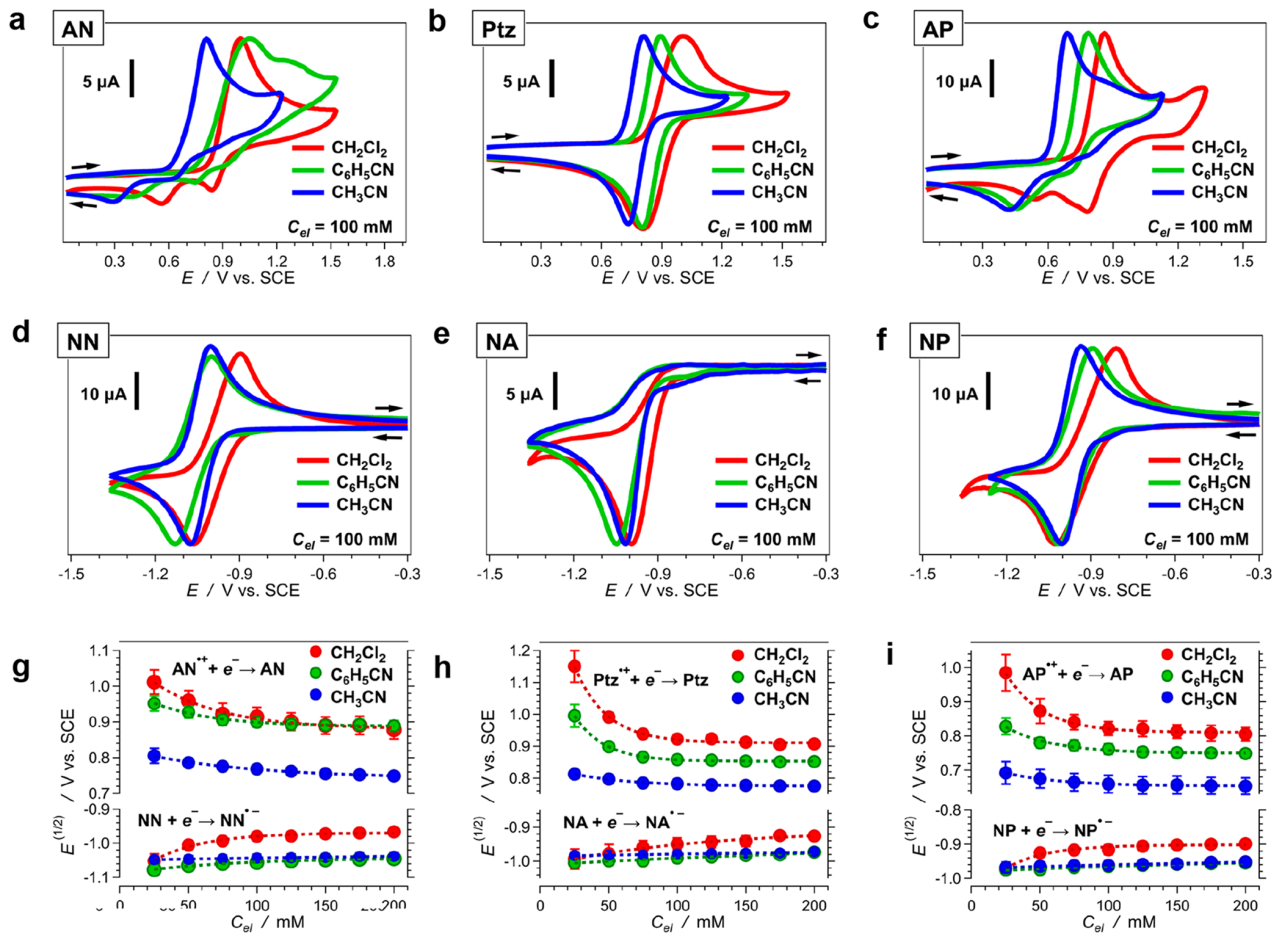
Dependence of the electrochemical properties of the donors and the acceptors on solvent and electrolyte concentration, *C*_*el*_. The electrolyte is (*n*-C_4_H_9_)_4_NPF_6_. (a–f) Cyclic voltammograms of AN, Ptz, AP, NN, NA, and NP (Figure 1) for different solvents in the presence of 100 mM electrolyte, recorded at scan rate, ν = 150 mV s^–1^. (g–i) Dependence of the reduction potentials of (g) the two-ring species, AN^•+^ and NN, (h) the three-ring species, Ptz^•+^ and NA, and (i) the four ring species, AP^•+^ and NP, on the electrolyte concentration for different solvents.

Varying the electrolyte concentration (*C*_*el*_) between 25 and 200 mM for dichloromethane (CH_2_Cl_2_), benzonitrile (C_6_H_5_CN), and acetonitrile (CH_3_CN), reveals key trends for the dependence of the electrochemical properties of the six analytes on the medium composition (Figure 2).

The cyclic voltammograms of Ptz show chemically reversible oxidation (Figure 2b). For the different solvents and *C*_*el*_, the reduction half-wave potentials (*E*^(1/2)^) spread between about 0.8 and 1.1 V vs SCE (Figure 2h). This behavior is consistent with single-electron oxidation of phenothiazine derivatives based on reported pulse-radiolysis and cyclic voltammetry studies.^44−46^

The bent ground-state structure of Ptz makes its nitrogen at position 10 quite susceptible to protonation. The p*K*_a_ values of protonated phenothiazines in aqueous media range between about 4 and 7,^47,48^ and acidic impurities in the Ptz samples may distort the appearance of its voltammograms.^49,50^ In addition, the propensity of analytes to aggregate upon adsorption on the working-electrode surface tends to perturb the shapes of their voltammograms and shift the estimated *E*^(1/2)^ values.^51^ In CH_3_CN, for example, Ptz appears to show such tendencies (see Supporting Information), which warrants another consideration, i.e., to keep the concentration of Ptz at about 3 mM in the solutions for the electrochemical studies.

Lowering sample concentration is also important for keeping the Faradaic currents relatively small and preventing deformation of voltammograms due to voltage drops across the electrodes. Such voltage drops are especially significant for nonpolar solvents and low electrolyte concentrations. The measured potentials correspond to the applied voltage differences, *E*_RW_, between the terminals of the working and the reference electrodes. Of interest for electrochemical analysis, on the other hand, is the potential difference across the double layer, *E*_F_, on the surface of the working electrode where the Faradaic processes occur. The principal difference between *E*_RW_ and *E*_F_ originates from the voltage drop between the electrodes across the electrolyte solutions with resistance *R*, i.e., *E*_RW_ ≈ *E*_F_ + *iR* where *i* is the measured current. Indeed, highly conductive electrolyte solutions, small sample concentrations, and a short distance between the reference and the working electrode keep *R* and *i* small, allowing the approximation of *E*_F_ to *E*_RW_. Furthermore, while this *iR* voltage drop affects kinetic analyses, it tends to have quite small effects on extracted thermodynamics information from voltammograms, especially from those that show reversibility.

**Scheme 1:**
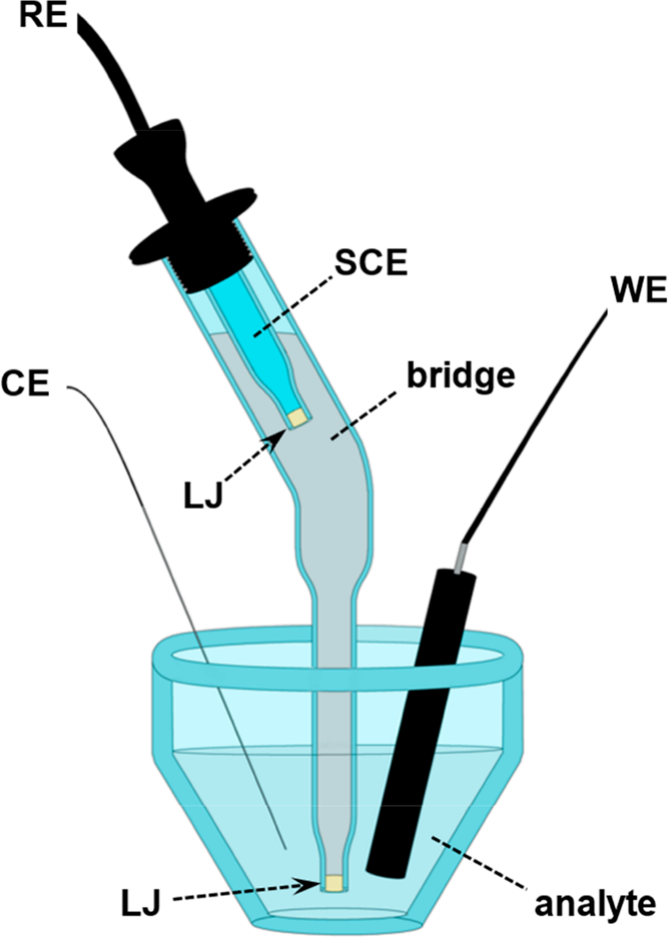
Three-Electrode Electroanalytical Cell with an SCE Reference Electrode Connected via a Salt Bridge RE—reference electrode; WE—working electrode; CE—counter electrode; LJ—liquid junction.

For acetonitrile solutions in the cells we employ (Scheme 1), the measured *R* varies between about 0.2 and 1 kΩ upon decreasing *C*_*el*_ from 200 to 25 mM. For dichloromethane, on the other hand, *R* exceeds 5 kΩ as *C*_*el*_ drops to 50 and 25 mM. For the nitrile solutions, as a result, changing sample concentration alters *E*_Ptz^•+^|Ptz_^(1/2)^ by less than *k*_*B*_*TF*^–1^, i.e., 26 mV. In contrast, lowering the concentration of Ptz in dichloromethane decreases the estimated *E*_Ptz^•+^|Ptz_^(1/2)^ values by more than 30 mV for *C*_*el*_ = 50 mM and by more than 100 mV for *C*_*el*_ = 25 mM. Because the anodic Faradaic currents are larger in magnitude than the cathodic ones even for reversible oxidation, the *iR*-induced positive shifts of the anodic peaks are larger than the negative shifts of the anodic ones, which is consistent with the observed behavior of *E*_Ptz^•+^|Ptz_^(1/2)^. Implementing *iR* compensation in the measurements can immensely improve the shapes of “distorted” cyclic voltammograms, decreasing the difference between the anodic and the cathodic peak potentials. For low sample concentrations, the changes in the estimated *E*_Ptz^•+^|Ptz_^(1/2)^ values that the *iR* compensation induces do not exceed 20 mV (see Supporting Information). Nonetheless, all potentials used for this study are extracted from voltammograms with implemented *iR* compensation.

As expected for the oxidation of noncharged species, the reduction potential of Ptz^•+^ increases with decreasing solvent polarity and electrolyte concentration (Figure 2h).^17^ Adding electrolytes to non-hydrogen-bonding solvents with relatively low polarity (1) introduces the conductivity needed for electrochemical measurements, and (2) increases their static dielectric constants.^10−12^ It is consistent with the similarities between the shifts in *E*_Ptz^•+^|Ptz_^(1/2)^ induced by solvent polarity and by *C*_*el*_. That is, for CT leading to a decrease in the charge of the analyte species, i.e., Ptz^•+^ → Ptz, the reduction potential decreases, i.e., showing negative shifts, with an increase in medium polarity.

Unlike that of Ptz, the electrochemical oxidation of AN and AP exhibits irreversibility (Figure 2a,c). Therefore, we extract the reduction potentials for their radical cations from the inflection points at the rises of the anodic waves. Such inflection-point potentials, *E*^(*i*)^, provide a good estimate of *E*^(1/2)^ for cyclic voltammograms showing irreversible behavior.^17^ Similar to that of Ptz, *E*^(1/2)^ (or *E*^(*i*)^) of AN and AP shows an increase with a decrease in solvent polarity and *C*_*el*_ (Figure 2g,i). The reduction potentials of AN^•+^ for benzonitrile and dichloromethane solutions, however, are quite similar, especially at high *C*_*el*_ (Figure 2g), despite the difference in polarity of these two solvents. This discrepancy warrants caution when correlating the bulk polarity of the solutions with the polarity at the electrode surfaces where the electrochemical CT occurs.

In contrast to the model electron donors, the voltammograms of the two- and four-ring acceptors, NN and NP, respectively, show reversible reduction, while the voltammograms of the three-ring NA reveal irreversibility (Figure 2d–f). These patterns of behavior are consistent with previously reported studies on the electrochemical reduction of these three nitroaromatics.^41,42,52^ Indeed, the values of *E*^(1/2)^ for the reduction of NN and NP represent the averages between the cathodic and anodic peak potentials, while *E*^(*i*)^ of the cathodic waves provides estimates for *E*^(1/2)^ of NA. For each solvent, the reduction potentials of the three nitroaromatics increase, i.e., show positive shifts, with an increase in *C*_*el*_ (Figure 2g–i). These findings are consistent with an electrolyte-induced increase in the polarity of organic solutions. An increase in medium polarity induces positive shifts in the reduction potentials that represent CT leading to an increase in the charge of the analyte species, i.e., N*X* → N*X*^•–^.

Nevertheless, the reduction potentials of NN, NA, and NP do not show the expected dependence on solvent polarity. For each of the nitroaromatics, the *E*^(1/2)^ values for the three solvents are quite close. Furthermore, the reduction potentials for the least polar dichloromethane tend to be slightly more positive than those for the nitrile samples (Figure 2g–i).

The Born solvation energy, Δ*G*_*B*_, of charged species describes well how medium polarity affects the reduction potentials, i.e.^53^
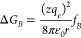
2awhere *z* is the charge and *r* is the radius of the solvated species, *q*_*e*_ is the elementary charge, ε_0_ is the vacuum permittivity, and *f*_*B*_ is the Born polarity function expressed in terms of the static dielectric constant, ε, of the medium:
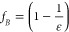
2b

The heterogeneous nature of electrochemical processes warrants caution in defining the polarity of the media surrounding the analyte species. Where needed, therefore, the discussion discerns between the polarity that molecular species experience in the bulk of the solution (*f*_*B*_^(*S*)^) and the polarity that species experience at the surface of the working electrode (*f*_*B*_^(*E*)^).

Indeed, an increase in *C*_*el*_ induces a decrease in the reduction potentials of the radical cations and an increase in the reduction potentials of the noncharged nitroaromatics (Figure 2g–i). These electrolyte-induced effects on *E*^(1/2)^ become more pronounced for less polar solvents (Figure 2g–i). Consideration of the inverse dependence of the solvation energy on the static dielectric constant (eq 2b) indicates that the same electrolyte-induced increase in a small ε should have a larger effect on (1 – ε^–1^) than in a large ε, e.g., changing ε from 10 to 20 results in a larger increase in *f*_*B*_ than changing it from 30 to 40.

Furthermore, the extent of the *C*_*el*_ effects on the *E*^(1/2)^ is quite similar for the three electron donors, AN, Ptz, and AP (Figure 2g–i). Because the Born solvation energy depends inversely on the radii of the redox species (eq 2a), this similarity in the *C*_*el*_ effects on *E*^(1/2)^ of the radical cations of the three donors suggests that they have similar effective radii, *r*_*eff*_, even though they comprise a different number of aromatic rings. Because none of AN, Ptz, and AP are spherical, the values of *r*_*eff*_ represent the radii of spherical ions with homogeneous charge distribution that experience the same solvation energy, Δ*G*_*B*_ (eq 2a), as the nonspherical AN^•+^, Ptz^•+^, and AP^•+^.^54^

The same argument can be extended to the reduction potentials of the three electron acceptors. Even though they comprise a different number of aromatic rings, the effects of *C*_*el*_ and the solvent on the measured *E*^(1/2)^ of NN, NA, and NP appear quite similar for all three of them (Figure 2g–i), indicating that their radical anions most likely have similar effective radii.

Based on eq 2, the lack of a strong dependence of reduction potentials on solvent polarity, as observed for the nitroaromatics, can originate from relatively large molecular sizes of the analytes. Nevertheless, the donors and the acceptors have similar structures, and while the reduction potentials of Ptz^•+^ and AP^•+^ distinctly show the expected dependence on solvent polarity, those of NA and NP do not. Considering that NA is planar and remains planar upon reduction, the oxidation-induced change in the Ptz geometry (Figure 1) can serve as a challenge for the presented argument based on similarity between the effective radii of Ptz^•+^ and NA^•–^. The structures of the naphthalene and pyrene derivatives and their radical ions, however, are all planar (Figure 1). In fact, the similar spreads of the spin-density distributions of AN^•+^ and NN^•–^, Ptz^•+^ and NA^•–^, and AP^•+^ and NP^•–^ (Figure 1) warrant negative shifts in *E*_A|A^•–^_^(1/2)^ that closely resemble the positive shifts in *E*_D^•+^|D_^(1/2)^ induced by the decrease in *C*_*el*_ and solvent polarity. It is contrary to what the experimental results show (Figure 2g–i). Therefore, the sizes of the nitroaromatics and their radical anions cannot account for the discrepancy in the polarity dependence of their reduction potentials.

For the electrochemical characterization of these analytes, we employ a three-electrode cell where the reference SCE electrode is connected to the analyte solution via a salt bridge. This configuration introduces two liquid junctions (Scheme 1): (1) between the saturated chloride aqueous solution (i.e., ≥4 M KCl) of the SCE electrode and the 0.1 M acetonitrile solution of (*n*-C_4_H_9_)_4_NPF_6_ in the bridge; and (2) between the electrolyte solution of the analyte and the bridge. Differences between the activities and mobilities of the ions on the two sides of the junctions produce liquid-junction potentials (*E*_LJ_), from the SCE-bridge junction, *E*_LJ_^(RB)^, and from the bridge-analyte junction, *E*_LJ_^(BE)^,^18^ i.e., *E*_LJ_ = *E*_LJ_^(RB)^ + *E*_LJ_^(BE)^. The values of *E*_LJ_ from the two junctions between the reference and working electrodes, therefore, are the same for all cathodic and anodic waves and shift the estimates of all reduction potentials, e.g., *E*_A|A^•–^_^(1/2)^ and *E*_D^•+^|D_^(1/2)^, in the same direction. The value of *E*_LJ_^(RB)^, originating from the interface between the SCE and the bridge, is the same for all measurements employing this cell setup. Conversely, *E*_LJ_^(BE)^ for the junction between the bridge and the analyte solution varies with changes in analyte solvent and *C*_*el*_. As a result, the bridge-analyte *E*_LJ_ can diminish, and even reverse, the observed solvent dependence of N*X* reduction potentials, while enhancing the solvent effects on *E*_D^•+^|D_^(1/2)^.

Resorting to differential reduction potentials, i.e., Δ*E*^(1/2)^(ε_E_) = *E*_D^•+^|D_^(1/2)^(ε_E_) – *E*_A|A^•–^_^(1/2)^(ε_E_), allows eliminating the effects of *E*_LJ_ while still obtaining reliable estimates for Δ*G*_PCT_^(0)^.^55^ Rearranging eq 1 allows expressing the driving force for medium with a dielectric constant ε in terms of Δ*E*^(1/2)^(ε_E_) for a solution with a dielectric constant ε_E_ used for measuring the reduction potentials:^2,55^
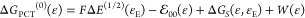
3a

For the validity of eq 3a, indeed, the reduction potentials of the acceptor and the oxidized donor i.e., *E*_A|A^•–^_^(1/2)^ and *E*_D^•+^|D_^(1/2)^ for estimating Δ*E*^(1/2)^, have to be obtained from voltammograms recorded under identical conditions in the same cell setup, i.e., ε_E_ = ε_D_ = ε_A_.^2^

The Born solvation term, Δ*G*_*S*_, in eq 3a assumes the following form:
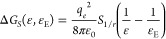
3bwhere *S*_1*/r*_ depends on the inverse radii of the donor and the acceptor, as well as on their initial charges, *z*_D_ and *z*_A_, and the number of transferred electrons, *n*_*e*_:
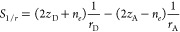
3c

For transfer of a single charge, *n*_*e*_ = 1, between an electroneutral donor and acceptor, *z*_D_ = *z*_A_ = 0, the expression for *S*_1*/r*_ simplifies to^55^
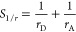
3d

This formalism also provides a relationship between differential reduction potentials for media with different polarities, which can prove tremendously useful for electrochemical analyses:^2,55^
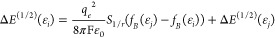
3e

**Figure 3:**
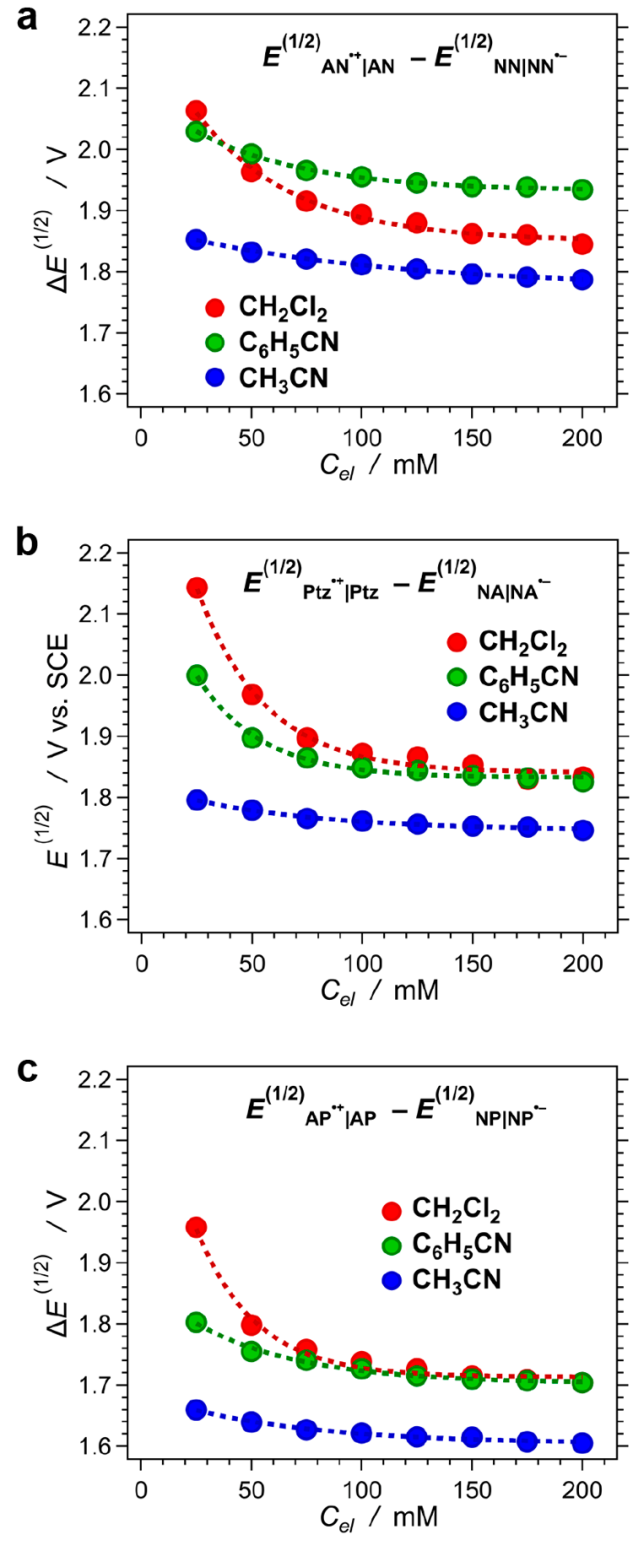
Dependence of the donor–acceptor differential potentials, i.e., Δ*E*^(1/2)^ = *E*_D^•+^|D_^(1/2)^ – *E*_A|A^•–^_^(1/2)^, on the concentration of the supporting electrolyte, *C*_*el*_, for dichloromethane, benzonitrile, and acetonitrile, for donors and acceptors comprising (a) two aromatic rings, i.e., naphthalene derivatives, (b) three aromatic rings, i.e., species with an anthracenyl skeleton, and (c) four aromatic rings, i.e., pyrene derivatives.

For each solvent, the differential reduction potentials for AN^•+^ and NN, Ptz^•+^ and NA, and AP^•+^ and NP, i.e., Δ*E*^(1/2)^ = *E*_D^•^|D_^(1/2)^ – *E*_A|A^•–^_^(1/2)^, decrease with an increase in *C*_*el*_ (Figure 3). This trend is more pronounced for the low-polarity solvent, i.e., CH_2_Cl_2_, than for the two nitriles (Figure 3).

A function that shows an asymptotic decrease to a value at large electrolyte concentrations, i.e., Δ*E*_*C*_*el*_→∞_^(1/2)^, can represent the dependence of Δ*E*^(1/2)^ on *C*_*el*_:
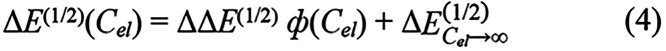
4where ΔΔ*E*^(1/2)^ is the maximum possible variation of Δ*E*^(1/2)^, between its value for a neat solvent, Δ*E*_*C*_*el*_ = 0_^(1/2)^, and Δ*E*_*C*_*el*_ → ∞_^(1/2)^, i.e., ΔΔ*E*^(1/2)^ = Δ*E*_*C*_*el*_ = 0_^(1/2)^ – Δ*E*_*C*_*el*_ → ∞_^(1/2)^. The function 
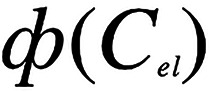
 varies between 1 and 0, i.e., 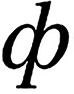
(0) = 1 and 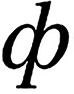
(∞) = 0. An exponential function, 
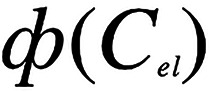
 = exp(− *γ C*_*el*_), with an empirical parameter γ provides a means for describing the dependence of Δ*E*^(1/2)^ on *C*_*el*_ for the analytes in the three solvents (Figure 3).

An increase in the donor–acceptor differential potential decreases the CT driving force, i.e., induces a positive shift in *G*_PCT_^(0)^ (eq 3a). For each of the donor–acceptor pairs, the differential potentials for acetonitrile are smaller than those for dichloromethane (Figure 3), which is consistent with enhancing the propensity for CS with increasing medium polarity.^1^ The differential potentials for benzonitrile, however, do not quite follow this trend. For three-ring and four-ring donor–acceptor pairs, an increase in *C*_*el*_ makes Δ*E*^(1/2)^ values for benzonitrile similar to those for dichloromethane (Figure 3b,c). This discrepancy is even more pronounced for the donor–acceptor pair of the naphthalene derivatives where Δ*E*^(1/2)^ for benzonitrile becomes larger than that for dichloromethane when *C*_*el*_ exceeds 50 mM (Figure 3a). These results indicate that in different solvents, the supporting electrolyte affects the medium polarity at the electrode surfaces to a different extent.

The striking similarity between eq 3e and 4 reveals a relationship between the medium polarity, *f*_*B*_^(*E*)^, which Ptz and NA experience on the surface of the working electrode, and the bulk electrolyte concentration *C*_*el*_:
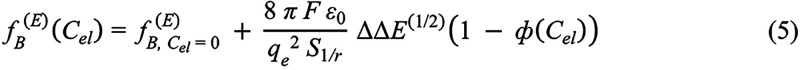
5

The Born polarity of the bulk of neat solvents, i.e., *f*_*B*,*C*_*el*_ = 0_^(*S*)^, is easy to determine from the readily available information about their static dielectric constants. Nonetheless, estimating the solvent polarity *f*_*B*,*C*_*el*_ = 0_^(*E*)^ at electrode surfaces with applied voltage potentials is not trivial. The huge electric fields, to which the solvent molecules are exposed at the liquid interface with the electrode, lead to *electrofreeze*.^16^ It involves alignment of the dipoles of the solvent molecules with the local electric field, acting against the entropy that randomizes the molecular order of the liquid. This field-induced alignment impedes the rotation of the solvent dipoles and, thus, decreases the orientational polarization and the dielectric constant of the microenvironment at the vicinity of the electrode.

The decreased dielectric constant of water in the presence of electric field, Π, can be estimated from its static dielectric constant, ε, refractive index, *n*, and dipole moment, μ: ε(Π) = *n*^2^ + 3(ε_Π=0_ – *n*^2^)(*βΠ*)^−1^L(*βΠ*), where *L*(*x*) = coth(*x*) – *x*^–1^ and =5μ(*n*^2^ + 2)(2*k*_*B*_*T*)^−1^.^56^ Although this expression, derived from the Onsager solvation model, appears to be valid also for organic solvents under applied fields,^16^ it presents two key challenges. (1) Experimentally, we control the potential of the working electrode and do not have a good handle on monitoring the exact field strength that the analyte species in the double layer experience. (2) It is challenging to estimate where exactly the analyte molecules are in the double layer unless they are immobilized on the electrode surface, proverbally via relatively rigid linkers. Using the experimentally obtained Δ*E*^(1/2)^ for the donor–acceptor pairs, therefore, we calculate *f*_*B*_^(*E*)^ – *f*_*B,C*_*el*_ = 0_^(*E*)^, rather than *f*_*B*_^(*E*)^, for the different electrolyte concentrations in the three solvents (eq 5). A global fit of the reduction potentials, *E*^(1/2)^, of the six compounds vs *f*_*B*_^(*E*)^ – *f*_*B,C*_*el*_ = 0_^(*E*)^, where *f*_*B,C*_*el*_ = 0_^(*E*)^ is one of the fitting parameters, allows estimating *f*_*B*_^(*E*)^ for the different electrolyte solutions.

**Figure 4:**
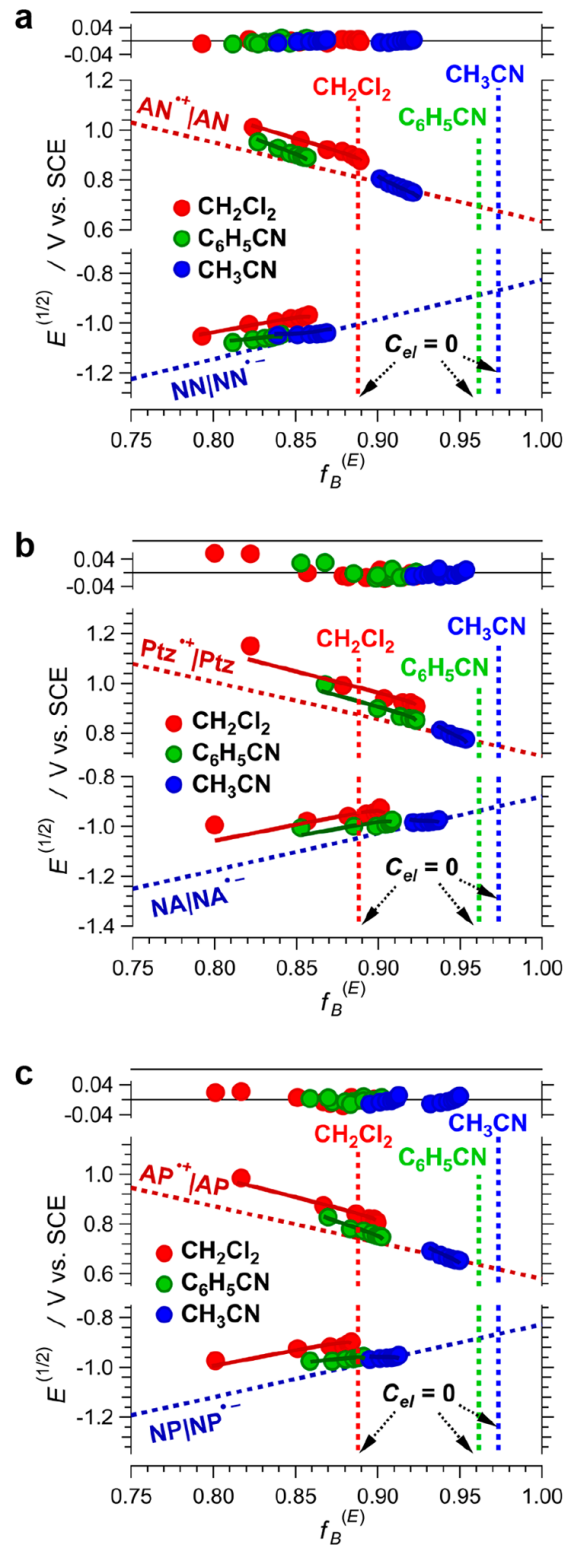
Dependence of the reduction potentials, *E*^(1/2)^, of the acceptors and the oxidized donors on the medium polarity at the electrode surface, *f*_*B*_^(*E*)^, obtained using eq 5. The solid lines represent global fits with the residuals shown on the top of the graphs. A linear function of *E*^(1/2)^ vs *f*_*B*_^(*E*)^, modified by adding *E*_LJ_^(*s*)^ and *E*_LJ_^(*el*)^, is used for the simultaneous fits of the 18 sets of data, i.e., of the six redox species in the three different solvents, where the polarity at zero-electrolyte concentration, *f*^(E)^_B,*Cel*=0_ (eq 5), is introduced as a fitting parameter (see Supporting Information for details). For each of the donor–acceptor pairs, the slopes, *S* = 0.5*S*_1*/r*_(8*πFε*_0_)^−1^, for the linear correlations between *E*^(1/2)^ and *f*_*B*_^(*E*)^ (i.e., of the dashed lines) are held the same for all solvents but with opposite signs for D^•+^|D and A|A^•–^. The slopes from this global fit analysis yield effective radii of (a) 4.5 Å for the naphthalene derivatives; (b) 4.9 Å for the derivatives with the anthracenyl skeleton; and (c) also 4.9 Å for the pyrene derivatives. The *E*_LJ_^(*s*)^ values are held the same for each solvent. For the acetonitrile samples, *E*_LJ_^(*s*)^ is kept at zero and allowed to optimize for the other two solvents. *E*_LJ_^(*el*)^ assumes the format of the Henderson equation, i.e., α ln(*C*_*el*_*/C*_*el*_^(*bridge*)^),^57^ where *C*_*el*_^(*bridge*)^ is the electrolyte concentration in the bridge and set at 100 mM, and α is introduced as a fitting parameter, held the same for all 18 sets of data. The global fit yields *E*_LJ_^(*s*)^ = 89 mV for the dichloromethane samples, and *E*_LJ_^(*s*)^ = 31 mV for the benzonitrile samples; and *E*_LJ_^(*el*)^ that varies between 21 and −11 mV for increasing *C*_*el*_ from 25 to 200 mM. The vertical dashed lines represent the *f*_*B*_ values for the three neat solvents. The linear components of the global-fit function, i.e., *S* and *E*^(1/2)^(*f*_*B*_^(E)^ = 0), allow estimating the reduction potentials for any other solvent medium with different polarity (Table 1).

Plots of the reduction potentials of the six compounds versus the thus obtained *f*_*B*_^(*E*)^(*C*_*el*_) values show linear correlations (Figure 4), where (1) the slopes for the oxidation of the donors and the reduction of the acceptors are the same but with opposite signs, which is consistent with their similar sizes, i.e., *r*_D^•+^_ ≈ *r*_A^•–^_ ≈ 2*S*_1/*r*_^–1^; and (2) the reduction potentials for CH_2_Cl_2_ are positively shifted in comparison with those for benzonitrile, which are positively shifted in comparison with those for acetonitrile (Figure 4). These patterns are consistent with added *E*_LJ_ originating from the junction between the bridge and analyte solutions (Scheme 1). The liquid-junction potential originates from the difference in the solvents, *E*_LJ_^(*s*)^, and the activity gradient of the electrolyte ions, *E*_LJ_^(*el*)^, across the junction. The additive of *E*_LJ_^(*s*)^ and *E*_LJ_^(*el*)^ can approximately represent the total *E*_LJ_.^18^

The global fit for all reduction potentials vs *f*_*B*_^(*E*)^, while considering *E*_LJ_ ≈ *E*_LJ_^(*s*)^ + *E*_LJ_^(*el*)^ and *r*_Ptz^•+^_ ≈ *r*_NA^•–^_ ≈ 2 *S*_1*/r*_^–1^, reveals that the solvent difference contributes principally to the observed *E*_LJ_. Interfacing the CH_2_Cl_2_ samples with the CH_3_CN bridge solution induces about a 90-mV positive shift in *E*^(1/2)^, and *E*_LJ_^(*s*)^ for the junctions between C_6_H_5_CN and CH_3_CN amount to about 30 mV. Conversely, *E*_LJ_^(*el*)^ varies between 22 and −11 mV for *C*_*el*_ from 25 to 200 mM (Figure 4). These small values for *E*_LJ_^(*el*)^ are consistent with the reported *E*_LJ_^(*el*)^ of only a few tens of mV originating from an order-of-magnitude difference between ion activity across a liquid junction.

These findings show that liquid junctions between solutions containing the same electrolyte with concentration differences smaller than a factor of 5 tend to generate *E*_LJ_^(*el*)^ that is smaller than the thermal potential, i.e., *k*_*B*_*TF*^–1^, which realistically often is within the experimental uncertainty of electrochemical measurements. That is, the principal contribution to *E*_LJ_ originates from differences between the solvents on the two sides of the junction, which affect the activity gradients and the mobilities of the present ions. Using the same electrolyte for the bridge and the analyte solutions and keeping a relatively small difference between their concentrations ensures negligibly small *E*_LJ_^(*el*)^ values. In such cases, therefore, variations in the analyte solvents represent the principal cause for the observed effects from the liquid-junction potential.

Another important implication of the outcomes from the global-fit analysis is the polarity *f*_*B*_^(*E*)^ that the different species experience at the electrode surface during reduction and oxidation. For the dichloromethane solutions, it ranges from about 0.8 and 0.9 (Figure 4), which corresponds to a static dielectric constant between 5 and 10, i.e., equal to or about twice smaller than dielectric constant of the neat solvent.

For the benzonitrile samples, *f*_*B*_^(*E*)^ overlaps with the *f*_*B*_^(*E*)^ values for the dichloromethane ones. For the naphthalenes in benzonitrile, *f*_*B*_^(*E*)^ is between 0.8 and 0.85 corresponding to narrowly distributed ε between 5 and 7; and for the three- and four-ring analytes, ε ranges from about 7 and 10 (Figure 4). These ε values are about 2.5 to 5 times smaller than the static dielectric constant of neat benzonitrile, which suggests a substantial field effect on this solvent comprising reactively bulky aromatic molecules with large dipoles making it quite susceptible to *electrofreeze*.

The *f*_*B*_^(*E*)^ for the acetonitrile samples is also consistent with field-induced *electrofreeze*, showing polarities that correspond to dielectric constants between about 6 and 20. Overall, *f*_*B*_^(*E*)^ for the reduction processes is smaller than *f*_*B*_^(*E*)^ for the oxidation ones (Figure 4). This behavior can originate from differences in (1) the composition of the Helmholtz layers, and (2) the field strength in the double layer during oxidation and reduction. The Helmholtz layer consists of tetrabutylammonium ions for the negatively polarized reducing electrode surface, and of hexafluorophsphate ions for the oxidation reactions. The aliphatic chains of the cations in the Helmholtz layer can contribute to lowering the microenvironment polarity that the nitroaromatics experience during reduction. Conversely, the magnitudes of the negative potentials (vs the reference electrode) applied to the working electrode for reducing the nitroaromatics are slightly larger than the magnitudes of the positive potentials needed for driving the oxidation of the electron-rich species. Hence, the field strength, and the extent of the *electrofreeze*, at the surface of the working electrode during the reduction steps should be larger than during the oxidation ones.

For nitriles, the smallest redox species, i.e., the naphthalenes, appear to experience a less polar microenvironment at the electrode surface than the three-ring and four-ring analytes (Figure 4). In comparison with the three-ring and four-ring species, the naphthalenes could assume a distribution that is closer to the electrode surface where the fields are stronger and the *electrofreeze* more pronounced.

Overall, the polarities, *f*_*B*_^(*E*)^, that the aromatic analytes experience at the electrode surface during oxidation and reduction tend to be smaller than the bulk polarities of the neat solvents, *f*_*B,C*_*el*_ = 0_^(*S*)^. How does *f*_*B*_^(*E*)^, however, compare to the bulk polarity of the electrolyte solutions, *f*_*B*_^(*S*)^?

### Effect of Electrolyte Concentration on Solution Polarity

The analysis, employing eqs 3 to 5, relates the bulk electrolyte concentration, *C*_*el*_, with the Born polarity, *f*_*B*_^(*E*)^, of the microenvironment that analyte species experience in the Helmholtz and the diffused layer at the surface of the working electrode during the heterogeneous CT steps. With relevance to homogeneous CT, it is key to understand how *C*_*el*_ affects the polarity, *f*_*B*_^(*S*)^, that the donor and acceptor species experience in the bulk of the electrolyte solutions, i.e., away from the electrode surface.

An optical approach, as implemented by the Lippert–Mataga–Ooshika (LMO) formalism, allows for extracting information about bulk medium polarity from the Stokes’ shifts,  of fluorescent chromophores:^58−60^
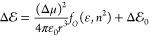
6awhere Δμ is the difference between the magnitudes of the dipoles of the emissive excited state (μ*) and the ground state, (**μ**_0_), i.e., Δμ = |μ*| – |**μ**_0_|;  is the Stokes’ shift for nonpolar media with equal static and dynamic dielectric constants, i.e., ε = *n*^2^; and *f*_*O*_(ε, *n*^2^) is the Onsager polarity function, representing the difference between the static, *f*_*O*_(ε), and the dynamic, *f*_*O*_(*n*^2^), reaction-field contributions^61^
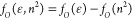
6bHere, for each solvent bulk characteristic, 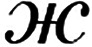, that can be ε or *n*^2^, the expression for the Onsager reaction-field function is^61^
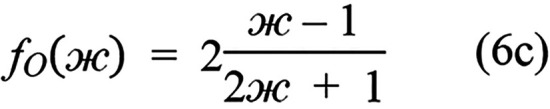
6cDepending solely on the static dielectric constant, ε, the Onsager static reaction-field term, *f*_*O*_(ε), in eq 6b relates to the Born polarity function, *f*_*B*_ (eq 2b):
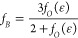
7a
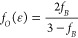
7b

The LMO analysis does not account for changes in dipole orientation, and a principal assumption is that μ* and **μ**_0_ are parallel and point in the same direction, i.e., Δμ = |Δ**μ**|, where |Δ**μ**| is the magnitude of the vector difference between the two dipole moments. That is, |Δ**μ**| = |μ* – **μ**_0_| or |Δ**μ**|^2^ = |μ*|^2^ + |**μ**_0_|^2^ – 2 |μ*| |**μ**_0_| cos(α), where α is the angle between the ground- and excited-state dipoles. Estimating the angle between the μ* and **μ**_0_ requires alternative methods.^62,63^

In general, the extent to which a solvent damps permeation of electric field correlates with its polarity. This medium polarization (**P**), which counters the field permeation, encompasses:^64^ (1) orientational polarization (**P**_**μ**_), originating from aligning the medium dipoles along the electric fields, which has a relatively slow response (>10 ps); (2) nuclear polarization (**P**_ν_), resulting from the shift of the medium nuclei toward the negative pole of the field, and has a slightly faster response (≳1 ps); and (3) electronic polarization (**P**_*e*_), arising from the femtosecond and subfemtosecond shifts of the electron density of the medium toward the positive pole of the field. All three forms of solvent polarization, **P**_**μ**_, **P**_ν_, and **P**_*e*_, contribute to its static dielectric constant, ε. Conversely, only the electronic polarization, **P**_*e*_, of a medium contributes to its dynamic dielectric constant, *n*^2^. Thus, *f*_*B*_ and *f*_*O*_(ε) encompass contributions from **P**_**μ**_, **P**_ν_, and **P**_*e*_, and *f*_*O*_(ε, *n*^2^) accounts only for **P**_**μ**_ and **P**_ν_ (eqs 2b, 6b and 6c). Originating from the Born model, the Pekar factor, γ = (*n*^–2^ – ε^–1^),^65^ also accounts only for the contributions from **P**_**μ**_ and **P**_ν_ to the solvation energy of ions and it is a key in the Marcus expression for the medium reorganization energy.^66^ These differential polarity functions, *f*_*O*_(ε, *n*^2^) and γ, reflect the notion that the optical-frequency solvent modes, responsible for *f*_*O*_(*n*^2^) and (1 – *n*^–2^), are fast enough to follow electronic transitions of solvated systems. Conversely, orientational and nuclear medium reorganization affect the dynamics of the solvated systems relaxing to equilibrium.

For polar solvents with ε ≳ 5, *n*^–2^ ≫ ε^–1^ and *f*_*O*_(*n*^2^) > *f*_*O*_(ε). It indicates that their *f*_*O*_(ε, *n*^2^) and γ depend predominantly on their refractive indices. This notion of *n*^2^ dominating *f*_*O*_(ε, *n*^2^) and γ may suggest that predominantly solvent polarizability governs the solvation dynamics of polar media. Detailed analysis, however, shows that the effects of medium polarizability on the reorganization energy are not as large as this train of thought may suggest.^67^ Conversely, the Born and Onsager models do not inherently account for optical-frequency solvent modes and the solvation dynamics.^68,69^ Furthermore, decoupling polarizability of a solvent from its overall polarity is not quite trivial. Nevertheless, within the scope of the utility of the Born and Onsager models, considering that ε represents cumulatively the effects of **P**_**μ**_, **P**_ν_, and **P**_*e*_ (that act in different time scales) and subtracting *f*_*O*_(*n*^2^) and (1 – *n*^–2^) from *f*_*O*_(ε) and *f*_*B*_, respectively, leaves **P**_**μ**_ and **P**_ν_ to govern the solvation of dipolar and charged species. Another important consideration that originates from these relationships is that the dependence of *E*^(1/2)^ on medium polarity becomes truly apparent for solvents with relatively low ε. While the *E*^(1/2)^ values for the nitrile solutions appear to “cluster” together, those for CH_2_Cl_2_ show distinctly informative trends (Figure 4).

As discussed, the LMO formalism can yield information about medium polarity, *f*_*O*_(ε, *n*^2^), originating solely from **P**_**μ**_ and **P**_ν_. Via *f*_*B*_, on the other hand, electrochemical analysis accounts for contributions from **P**_**μ**_, **P**_ν_, and **P**_*e*_. To relate *f*_*B*_ with *f*_*O*_(ε, *n*^2^), we resort to optical measurements of the indices of refraction, *n*, of the electrolyte solutions for independent estimates of *f*_*O*_(*n*^2^) (eqs 6b and 6c).

The LMO analysis utilizes molecular species as probes for solvent polarity. The ubiquitously used dielectric constant, ε, and index of refraction, *n*, represent the average response of a large volume of medium to permeating electric fields. For solvent mixtures, however, these averaged ε and *n* may fall short in representing what solvated charged and dipolar species experience. The distribution and the structure of the medium around the solvation cavity differs from its bulk. Therefore, while implicit implementation of a neat solvent with its ε and *n* can be a good approximation for accounting solvation effects, employing mixed media may warrant explicit introduction of its molecular-level interactions, which can be a daunting challenge.

As enormous as the electric fields from molecular dipoles are, they do not permeate more than a few nanometers into the surrounding polar media,^70^ warranting caution when employing bulk quantities of solvent mixtures for characterizing localized effects. These considerations become even more important for electrolyte solutions where the conductivity, contributing to the imaginary component of the dialectic constant, is prevalent.^71^ Therefore, using molecular probes for solvent polarity provides information for the local environment around the probe, validating its applicability to analysis of reduction and oxidation at nanometer scales.

**Figure 5:**
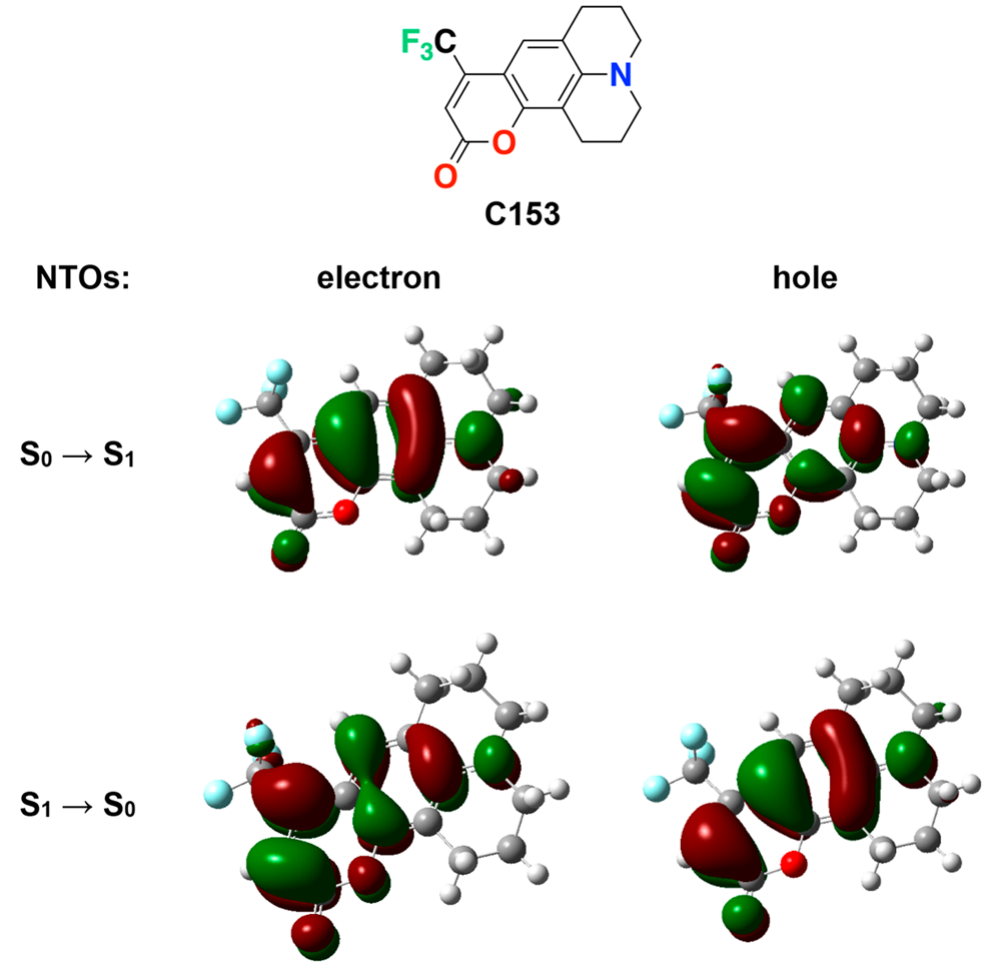
Electronic characteristics of coumarin 153 (C153) obtained from the Hartree–Fock (HF) framework employing the 6-311++G(2dF,3p) basis set, and configuration-interaction-singles (CIS) method for the excited states. The natural-transition orbitals (NTOs) are for CH_3_CN, where the solvent is implemented using the integral equation formalism variant of the polarizable continuum model (IEFPCM). The difference between the electric dipoles of the optimized excited and ground states, Δμ, is 3.07 D for CH_2_Cl_2_, 3.38 D for C_6_H_5_CN, and 3.45 D for CH_3_CN.

A chromophore that has a fluorescent excited state with a pronounced CT character ensures the large Δμ needed for estimating *f*_*O*_(ε, *n*^2^) of the electrolyte solutions using eq 6a. Also, a principal assumption for the validity of eq 6a is that medium polarity has a relatively small effect on Δμ. As an amino derivative of an electron-deficient aromatic dye, coumarin (C153) forms a CT excited state with μ* that is significantly larger than its ground-state dipole, **μ**_**0**_, and it has served as a photoprobe for a range of studies on solvation response.^69,72^ The julolidine structure of C153 (Figure 5) suppresses the dihedral rotation of the amine and prevents the formation of a twisted intramolecular charge-transfer (TICT) state, which not only can lead to emission quenching, but also to a noticeable dependence of μ* on solvent polarity. Computational analysis reveals that (1) the angle between **μ**_**0**_ and μ* of C153 is negligibly small and varies between 1.3° and 1.4° for the CH_2_Cl_2_, C_6_H_5_CN, and CH_3_CN; and (2) Δμ of C153 increases from 3.07 D for CH_2_Cl_2_ to 3.46 D for CH_3_CN (see Supporting Information). This Δμ increase, induced by medium polarity, is only about 12%, and is consistent with more pronounced enhancement of μ* than of **μ**_**0**_, originating from the Onsager reaction field in the solvated cavity.^64^ Therefore, C153 proves to be a good selection for the LMO analysis of the polarity of the electrolyte solutions.

**Figure 6:**
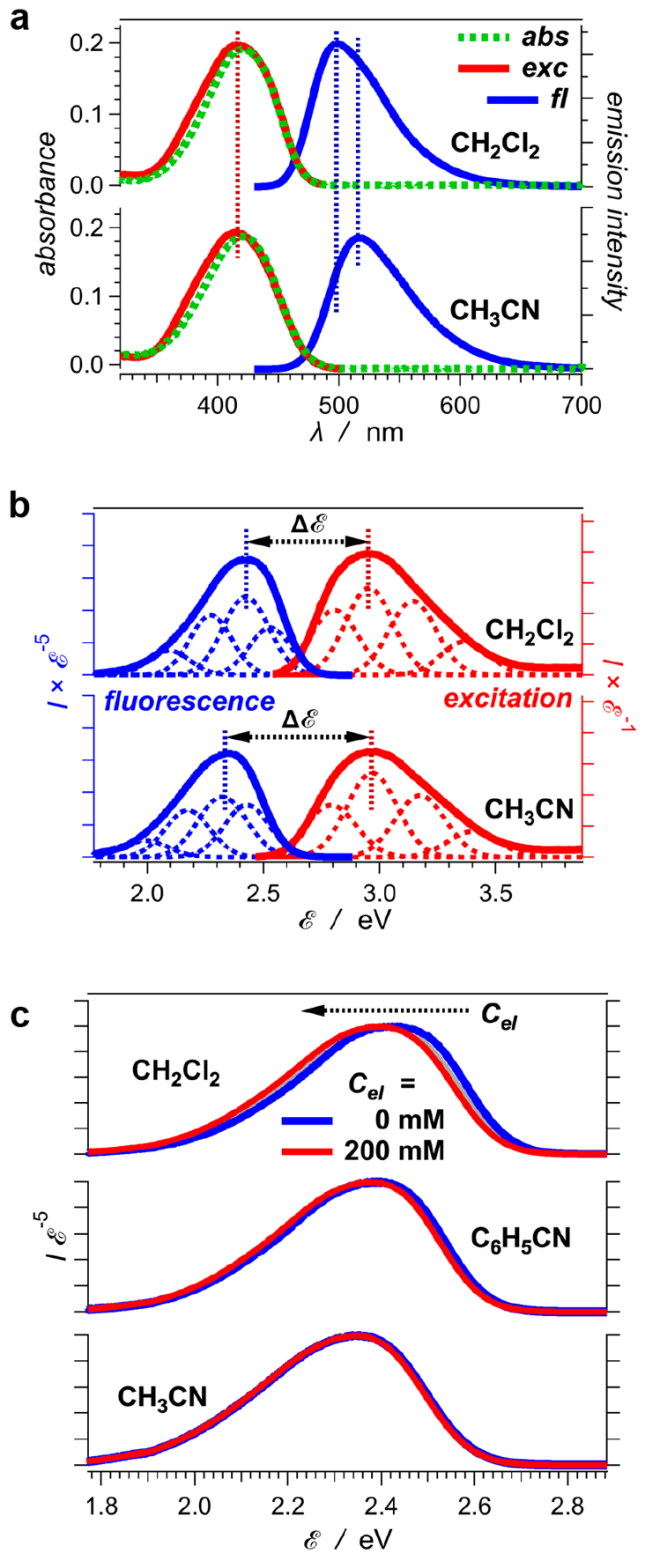
Steady-state optical spectra of coumarin 153 (C153). (a) Absorption, fluorescence, and excitation spectra for neat CH_2_Cl_2_ and CH_3_CN. (b) Fluorescence and excitation spectra for neat CH_2_Cl_2_ and CH_3_CN, plotted against energy scale on the abscissa, with transition-dipole-moment (TDM) correction applied to their intensity, *I*.^73^ The TDM corrections tend to “flatten” the peaks and introduce uncertainty in estimating the energy values of the spectral maxima. Therefore, the spectral maxima, needed for calculating the Stokes’ shifts, , are extracted from fits to a sum of Gaussians. (c) Effect of electrolyte concentration, *C*_*el*_, on the fluorescence spectra of C153 for CH_2_Cl_2_, C_6_H_5_CN, and CH_3_CN. The spectra for no electrolyte and 200 mM electrolyte are shown in blue and red, respectively, and the spectra for the other electrolyte concentrations (between 25 and 175 mM) are shown in gray. (For the emission spectra, λ_*ex*_ = 410 nm. For the excitation spectra, λ_*em*_ = 500 nm for CH_2_Cl_2_, and λ_*em*_ = 510 nm for C_6_H_5_CN and CH_3_CN.)

**Figure 7:**
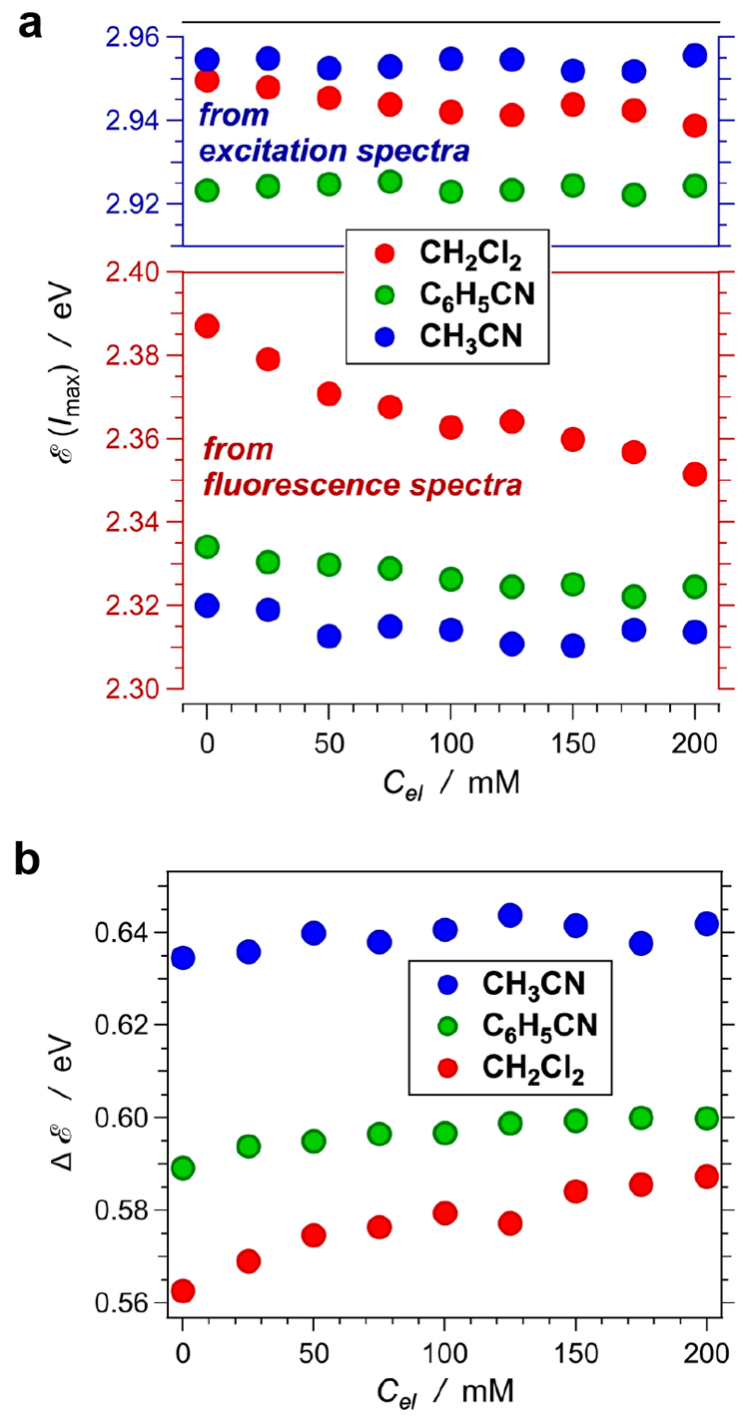
Effects of solvents and electrolyte concentration, *C*_*el*_, on (a) the position of the spectral maxima, , and (b) the Stokes’ shift, , of coumarin C153 (Figure 6b).

The solvent and *C*_*el*_ affect the emission properties of C153 more strongly than its absorption (Figures 6 and 7a). Furthermore, the *C*_*el*_-induced spectral shifts are more pronounced for the CH_2_Cl_2_ solutions than for the nitrile ones (Figures 6c and 7a). As expected, an increase in solvent polarity and *C*_*el*_ enhances the Stokes’ shift of C153 (Figure 7b). A linear fit of  for neat solvents vs their Onsager polarity provides (Δμ)^2^*r*^–3^ and  for C153 (eq 6a). Implementing these results in eq 6a yields estimates of *f*_*O*_(ε, *n*^2^) for the different *C*_*el*_ from the Stokes’ shifts of C153 in the electrolyte solutions (Supporting Information).

Concurrent measurements of the refractive indices of the electrolyte solutions produce the dynamic-dielectric component of the Onsager function, *f*_*O*_(*n*^2^) (eqs 6b and 6c). The added electrolyte induces negligible perturbations to the refractive indices of the neat solvents. That is, the polarizability and the electronic polarization, **P**_*e*_, of the component ions, (*n*-C_4_H_9_)_4_N^+^ and PF_6_^–^, are comparable to those of the three solvents. Specifically, an increase in *C*_*el*_ slightly raises *f*_*O*_(*n*^2^) of the CH_3_CN solutions, while lowering *f*_*O*_(*n*^2^) of the C_6_H_5_CN samples. The cumulative polarizability of the electrolyte ions appears to be on par with that of CH_2_Cl_2_ (Supporting Information).

**Figure 8:**
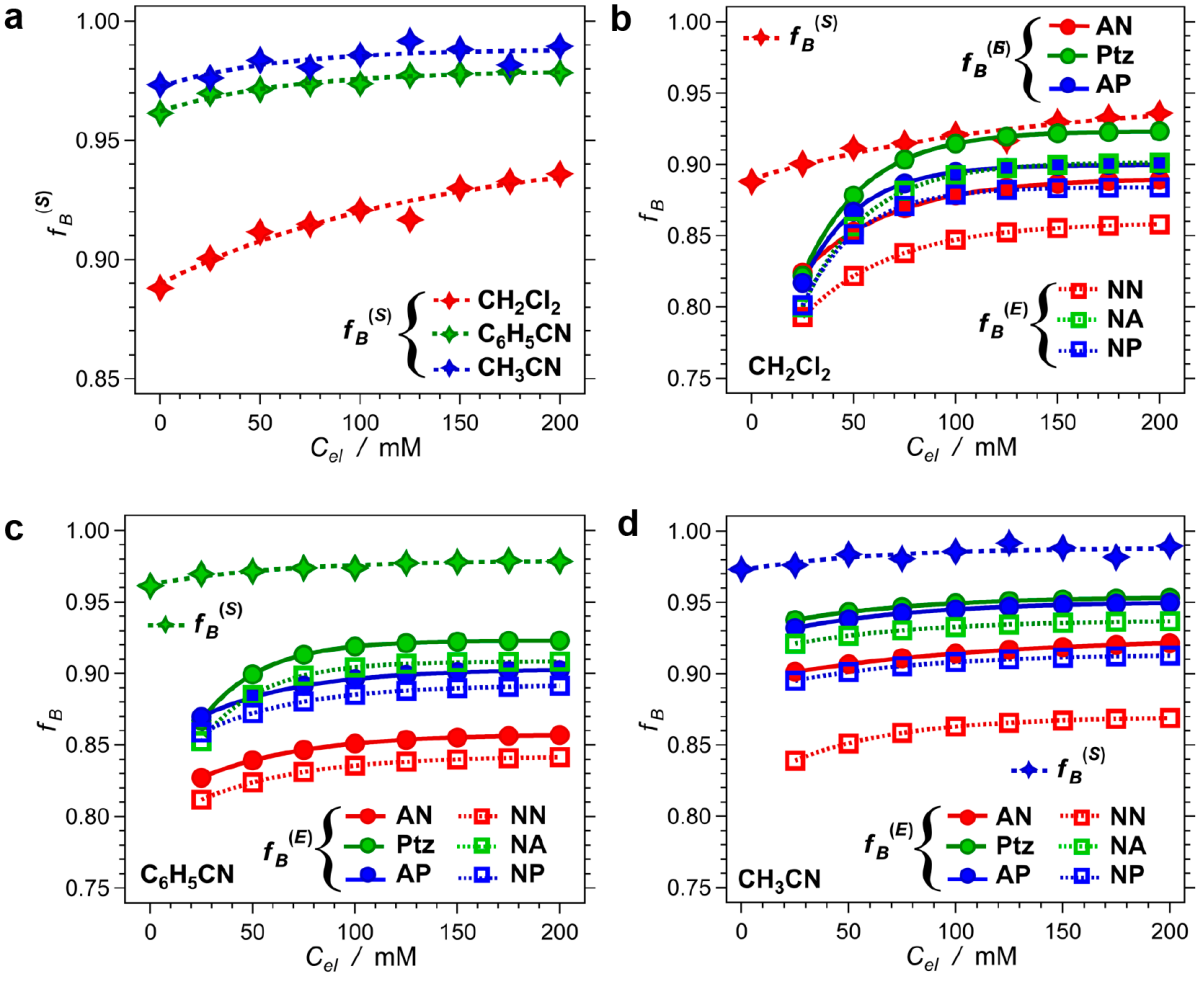
Dependence of the Born polarity, *f*_*B*_, on electrolyte concentration, *C*_*el*_. (a) Dependence of the bulk-solution polarity, *f*_*B*_^(*S*)^, obtained from the LMO analysis of C153, on *C*_*el*_ of the electrolyte solutions in dichloromethane, benzonitrile, and acetonitrile. (b–d) Comparison between the bulk solution polarity, *f*_*B*_^(*S*)^, and the polarity *f*_*B*_^(*E*)^ that the redox species experience on the surface of the working electrode obtained from eq 5 and the global-fit analysis of the reduction potentials (Figure 4).

The differences between *f*_*O*_(ε, *n*^2^) and *f*_*O*_(*n*^2^) yield *f*_*O*_(ε) that allow estimating the bulk Born polarity, *f*_*B*_^(*S*)^, of the electrolyte solutions (eq 7a). An increase in *C*_*el*_ induces an increase in *f*_*B*_^(*S*)^, which is consistent with the reported increases in the static dielectric constant (extracted from capacitance measurements) upon increasing *C*_*el*_ of organic solutions.^10−12^ This trend in *C*_*el*_ dependence of the bulk polarity, *f*_*B*_^(*S*)^, however, is not as pronounced as the *C*_*el*_-induced enhancement of the polarity, *f*_*B*_^(*E*)^, that the three donors and the three acceptors experience during oxidation and reduction at the electrode surface (Figure 8).

Furthermore, the medium polarity at the electrode surface is smaller than the polarity that molecular species experience in the bulk of the solutions (Figure 8b–d), which is consistent with the field-induced *electrofreeze*.

Adding electrolyte to the three organic solvents increases their polarities (Figure 8a). At the electrode surface, on the other hand, the applied field decreases the medium polarity. For polar solvents, the latter effect is more prevalent than the former (Figure 8b–d). For solvents with dielectric constants exceeding 10, i.e., *f*_*B*_ exceeding 0.9, an increase in ε by the added electrolyte does not cause much of an increase in *f*_*B*_. Conversely, the large dipoles of the molecules of polar solvents increase their susceptibility to the field-induced *electrofreeze*. As a result, the polarity of neat dichloromethane matches the polarity of electrochemical reduction and oxidation for most CH_2_Cl_2_ samples containing 50 to 200 mM electrolytes (Figures 4 and 8b). On the other hand, none of the electrolyte solutions of the two nitriles provides a microenvironment at the electrode surface that has the polarity of the neat solvents (Figure 4, 8c,d). For the polar solvents, a further increase in *C*_*el*_ will not significantly increase *f*_*B*_. The addition of electrolyte to about 100 mM accounts for most of the polarity enhancement, and any increase of *C*_*el*_ beyond 200 mM has a negligible effect on the *f*_*B*_. Quantifying these electrolyte effects on *f*_*B*_^(*E*)^ is essential for the reliable use of electrochemical data for the analysis of CT thermodynamics as eqs 1 and 3 implement.

## Discussion

The mismatch between the polarity of the medium surrounding donor–acceptor conjugates and the polarity that the electron donors and acceptors experience at the surfaces of the working electrodes during electrochemical analysis presents a key challenge for obtaining reliable estimates of CT driving forces (eqs 1 and 3). For AP^•+^, for example, the extrapolated reduction potential for bulk neat dichloromethane (Table 1) matches the measured *E*^(1/2)^ values, corrected for *E*_LJ_^(BE)^, for the same solvent in the presence of 75 to 100 mM electrolyte. For NP, the extrapolated neat-dichloromethane values of *E*^(1/2)^ match the electrochemically measured reduction potentials in the presence of 125 to 150 mM electrolyte. For the small-size AN^•+^, 125 mM electrolyte provides *E*^(1/2)^ that matches the extrapolated value for neat CH_2_Cl_2_. Nevertheless, for NN with the same small size, even at *C*_*el*_ = 200 mM the measured *E*^(1/2)^ is about 50 mV more negative than the extrapolated reduction potential for the neat CH_2_Cl_2_ (Figure 4a, Table 1).

**Table 1:** Reduction Potentials of the Six Redox Species in Neat Solvents, Extrapolated from the Global Fit Analysis (Figure 4)^a^

			*E*_D^•+^|D_^(1/2)^*/*V vs SCE			*E*_A|A^•–^_^(1/2)^*/*V vs SCE		
solvent^b^	ε^c^	*f*_*B*_^(*S*)^^d^	AN	Ptz	AP	NN	NA	NP
CH_3_CN	37.5	0.97	0.67	0.75	0.62	–0.87	–0.92	–0.87
C_6_H_5_CN	25.9	0.96	0.69	0.76	0.64	–0.89	–0.94	–0.88
CH_2_Cl_2_	8.93	0.89	0.81	0.87	0.74	–1.00	–1.05	–0.99
THF	7.58	0.87	0.84	0.90	0.77	–1.04	–1.07	–1.02
CHCl_3_	4.81	0.79	0.96	1.02	0.88	–1.16	–1.19	–1.13
C_6_H_5_CH_3_	2.38	0.58	1.30	1.33	1.19	–1.49	–1.50	–1.44
C_6_H_6_	2.27	0.56	1.33	1.36	1.22	–1.53	–1.53	–1.47
C_6_H_12_	2.02	0.50	1.42	1.44	1.30	–1.61	–1.61	–1.55

a Potentials extrapolated from the global-fit results for *S*_1*/r*_ and *E*^(1/2)^(*f*_*B*_^(E)^ = 0) (Figure 4), i.e., *E*^(1/2)^*= E*^(1/2)^(*f*_*B*_^(E)^ = 0) + *Sf*_*B*_^(*S*)^, where *S* = *S*_1*/r*_(16*πFε*_0_)^−1^ for reduction and *S* = −*S*_1*/r*_(16*πFε*_0_)^−1^ for oxidation, assuming single-electron processes involving species with equal sizes.

b Solvents: acetonitrile (CH_3_CN), benzonitrile (C_6_H_5_CN), dichloromethane (CH_2_Cl_2_), tetrahydrofuran (THF), chloroform (CHCl_3_), toluene (C_6_H_5_CH_3_), benzene (C_6_H_6_), cyclohexane (C_6_H_12_).

c Static dielectric constants.

d Values of the Born polarity function (eq 2b) for the neat solvents.

An increase in solvent polarity and the decrease in the molecular size of the analyte enhance these mismatches in the *E*^(1/2)^ values. For AP^•+^ and NP in acetonitrile, the difference between the extrapolated reduction potentials for the neat solvent and the measured *E*^(1/2)^ values in the presence of 200 mM electrolyte amount, respectively, to 40 and 90 mV (Figure 4b,c, Table 1). For AN^•+^ and NN in acetonitrile, these differences in *E*^(1/2)^ amount to about 70 and 170 mV, respectively.

Indeed, low-polarity solvents are not as favorable for electrochemical analysis as the polar ones. Nevertheless, employing electrolyte solutions in low-polarity solvents provides improved chances for matching the polarity of the neat solvents with the polarity that the analytes experience at the electrode surfaces. Also, an increase in analyte size and its effective radius decreases the polarity dependence of the measured potentials and the overall errors in estimating the CT driving forces.

The challenges of electrochemistry in low-polarity media, however, cannot be underestimated, especially for small electrolyte concentrations. Furthermore, the electrochemical windows of the electrolyte solutions present another constraint in the solvent selection. Because this analysis employs electron donor–acceptor pairs, it is important for the electrochemical windows of the solvents and their solutions to accommodate both the reduction of the donor and the oxidation of the acceptor.

Because an increase in the field strength at the electrode surfaces enhances the *electrofreeze* effects, analysis of donors and acceptors that are difficult to oxidize and reduce, respectively, enlarges the differences between *f*_*B*_^(*E*)^ and *f*_*B*_^(*S*)^. That is, electrochemical analysis of species that manifest Faradaic signals at low potentials warrants lesser discrepancies between *f*_*B*_^(*E*)^ and *f*_*B*_^(*S*)^.

Overall, estimating *f*_*B*_^(*E*)^ from electrochemical analysis of electron donors and acceptors in multiple solvents with various amounts of electrolyte, offers a means for introducing the measured reduction potentials, with the corresponding values of ε_D_, ε_A,_ and ε_E_, in eqs 1 and 3.

As prevalent as liquid junction potentials can be, the discussions about them are still few and far between. Measured electromotive forces (EMFs) for cells with different liquid junctions provide an experimental means for estimating the *E*_LJ_ values for interfaces between the employed solutions.^18,19,74,75^ The required specialized cells and equipment for sensitive EMF measurements render the evaluation of *E*_LJ_ impractical for laboratories equipped for “routine” electrochemical analysis, employing, for example, three-electrode setups (Scheme 1). Conversely, the lack of sufficient information about the mobility and the activity coefficients of ions composing electrochemical electrolytes (at mM concentrations in different solvents) presents challenges for obtaining theoretical estimates of *E*_LJ_. Therefore, despite the general awareness of the impacts that liquid junctions can have on electrochemical analysis, the lack of a facile means for evaluating *E*_LJ_ damps the enthusiasm for discussing this subject.

The values of *E*_LJ_ are usually embedded in the results from electrochemical analyses, and the common approach involves resorting to cell configurations with minimized *E*_LJ_. Indeed, keeping identical solutions, or at least the same solvents, on both sides of each junction, ensures relatively small *E*_LJ_, e.g., *E*_LJ_ < *k*_*B*_*TF*^–1^. Such confinement to analyte solutions that are similar to the media inside the salt bridges and the liquid reference electrodes, however, can prove quite limiting for the wealth of information that electrochemical analysis offers. Pseudoreference electrodes, which are in direct contact with the analyte solution, eliminate the liquid–liquid junctions from the cell setup. The potentials of such electrodes, however, depend on the media in which they are immersed leading to systematic errors and discrepancies between the results for different analyte solutions.

Using redox pairs with known potentials, such as ferrocenium|ferrocene, as internal standards, can provide a means to correct for the deviations that *E*_LJ_ introduces. Despite some early reports in the literature,^76−81^ however, solvent polarity and *C*_*el*_ affect the reduction potential of ferrocenium.^5,82^ The effects of medium polarity on the electrochemical potentials originate from the solvation energy, and their magnitude is inversely proportional to the effective radii of the species (eqs 2, 3). That is, the reduction potentials of large moieties, with charges homogeneously distributed over their whole structures, are considerably less susceptible to medium polarity than the reduction potentials of small species,^54^ such as ferrocenium.

Regarding analysis of CT thermodynamics (eq 1), utilizing differential potentials, Δ*E*^(1/2)^ (eq 3), eliminates the effects of *E*_LJ_.^2,55^ In fact, implementing in eq 1 reduction potentials of the acceptor and the oxidized donor measured in the same lab, with the same cell setup and the same electrolyte solution, is identical to using Δ*E*^(1/2)^, which for decades have provided reliable estimates of CT driving forces. The challenges, and potential errors, arise when imputting in eq 1 potentials measured with different cell setups under different conditions.

As invaluable as differential potentials are for analysis of CT thermodynamics, they also prove quite useful for estimating the polarity, *f*_*B*_^(*E*)^, that the redox species experience at the surface of the working electrode (eq 5). Assuming that *f*_*B*_^(*E*)^ at the surface of the polarized electrode is similar under the applied positive and negative biases, this information allows for estimating the *E*_LJ_^(BE)^ values for the junction between the bridge and the different analyte solutions (Scheme 1), and extracting reduction potentials for media with different polarity (Figure 4).

It is important to emphasize that even after these corrections for the bridge-analyte *E*_LJ_, the reported potentials vs SCE are actually vs SCE with added *E*_LJ_^(RB)^ from the junction between the bridge and the saturated KCl solution (Scheme 1). Using water-miscible solvents in the bridge is paramount for minimizing *E*_LJ_^(RB)^. SCE electrodes form some of the smallest *E*_LJ_ with alcohol solutions.^83^ From the aprotic solvents with wide electrochemical windows, CH_3_CN solutions form junctions with SCE with some of the smallest *E*_LJ_, i.e., not exceeding 0.1 V,^83^ making it a preferable solvent for the bridge media. Indeed, using CH_3_CN electrolyte solution for the bridge is practically ideal when Ag immersed in AgNO_3_ solution in CH_3_CN is used for a reference electrode.

Liquid-junction potentials can have substantial contributions to results from electrochemical analysis. Working with differential potentials, Δ*E*^(1/2)^, provides a means for eliminating the effects from *E*_LJ_. Actually, all reduction potentials are reported as potential differences from common reference electrodes, such as SCE and NHE. These reported values, however, commonly contain the *E*_LJ_ from the junctions between the reference electrodes and the media in which they are immersed during the measurements. Therefore, using such values from different sources warrants caution and understanding of the experimental setups.

## Conclusions

In electrochemical analysis, liquid-junction potentials appear to be “the elephant in the room” that is rarely discussed. Using differential potentials for donor–acceptor pairs allow eliminating *E*_LJ_ from the estimates of CT driving forces (eq 3). This approach, however, confines the utility of the measured reduction potentials only to the specific donors and acceptors that are analyzed with the same electrochemical setup under identical conditions. Furthermore, even the use of differential potentials, which eliminate the contributions from the liquid junctions, warrants a good understanding of the media polarity that the donor and acceptor analytes experience at the surfaces of the working electrodes. The invariance to *E*_LJ_ is a principal advantage of this differential-potential analysis. This invariance offers a relatively facile access to trends that reveal: (1) the effects of liquid-junction potentials and a means for estimating them, and (2) an approach for evaluating the polarity that redox species experience at the surfaces of polarized working electrodes (eq 5 and Figure 4). Therefore, global analysis of the electrochemical potentials of multiple donors and acceptors in a range of solvents with varying amounts of supporting electrolytes can prove crucial for exploring the frontiers of charge-transfer science.

## Supporting Information Available

Experimental section, providing details on the cyclic-voltammetry, the optical spectroscopy, the refractometry, and the computational analysis used for this study (PDF)

## Abbreviations

AN: 1-naphthylamine
ANA: 9-amino-10-nitroanthracene
AP: 1-aminopyrene
C153: coumarin 153
*C*_*el*_: electrolyte concentration
CIS: configuration interaction singles
CS: charge separation
CT: charge transfer
Stokes’ shift
ΔΔ*E*^(1/2)^: maximum possible variation of Δ*E*^(1/2)^ upon changing *C*_*el*_ from zero to infinity
DFT: density functional theory
ε: static dielectric constant or relative permittivity
ε_E_: dielectric constant used for measuring the reduction potentials
*E*^(0)^: standard electrochemical potential
*E*^(1/2)^: half-wave potential
zero-to-zero energy
*E*_F_: voltage difference across the double layer on the surface of the working electrode where the Faradaic processes occur
*E*_LJ_: liquid-junction potential
*E*_LJ_^(BE)^: bridge-analyte liquid-junction potential
*E*_LJ_^(*el*)^: liquid-junction potential due to the activity gradient of the electrolyte
*E*_LJ_^(RB)^: liquid-junction potential between the reference electrode and the bridge
*E*_LJ_^(*s*)^: liquid-junction potential originating from the difference in the solvents
*E*_RW_: voltage difference between the termini of the working and reference electrodes
ET: electron transfer
*f*_*B*_: Born polarity
*f*_*B*_^(*E*)^: Born polarity at the working-electrode surface
*f*_*B*_^(*S*)^: Born polarity in the bulk of the medium
(*C*_*el*_): function depicting the trend of dependence of Δ*E*^(1/2)^ on *C*_*el*_
*f*_*O*_: Onsager function
*f*_*O*_(ε): static component of the Onsager function
*f*_*O*_(ε, *n*^2^): Onsager polarity function
*f*_*O*_(*n*^2^): dynamic-dielectric component of the Onsager function
γ: Pekar factor
GCS: Gouy–Chapman-Stern
Δ*G*_S_: Born-solvation-energy term correcting for the differences in the media where the reduction potentials of the acceptor and the reduced donor are measured and where the CT between the donor and the acceptor occurs
Δ*G*_B_: Born solvation energy of an ion
HF: Hartree–Fock
*iR*: potential drop due to resistance of the electrolyte solution
IUPAC: International Union of Pure and Applied Chemistry
*k*_*B*_*T F*^–1^: potential corresponding to the thermal energy (at 298 K, this is equal to 25.7 mV)
LMO: Lippert-Mataga-Ooshika
μ: magnitude of electric dipole moment
**μ**_**0**_: electric dipole moment of ground state
**μ***: electric dipole moment of emissive excited state
*n*: index of refraction
*n*^2^: dynamic dielectric constant
NA: 9-nitroanthracene
*n*_*e*_: number of transferred electrons
NHE: normal hydrogen electrode
NTO: natural-transition orbital
NN: 1-nitronaphthalen
NP: 1-nitropyrene
**P**: medium polarization
PCT: photoinduced charge transfer
**P**_*e*_: electronic polarization
PET: photoinduced electron transfer
PHT: photoinduced hole transfer
Ptz: 10-methylphenothiazine
**P**_**μ**_: orientational polarization
**P**_ν_: nuclear polarization
*R*: electrical resistance
*r*: radius of solvated species
*r*_A_: radius of acceptor
*r*_D_: radius of donor
*S*_1*/r*_: wighted sum of inverse radii
SCE: saturated calomel electrode
SDD: spin-density distribution
TDM: transition dipole moment
Tha: thianthrene
TICT: twisted intramolecular charge-transfer
*W*: Coulomb work
*z*: ionic charge
z_A_: initial charge of acceptor
z_D_: initial charge of donor
