# Supplementary material for: How Do Liquid-Junction Potentials and Medium Polarity at Electrode Surfaces Affect Electrochemical Analyses for Charge-Transfer Systems?
Source: J Phys Chem B. 2023 Feb 3;127(6):1443–58. doi: 10.1021/acs.jpcb.2c07983 (PMC10234590; doi:10.1021/acs.jpcb.2c07983)
Supplement: Supplementary file 1 — jp2c07983_si_001.pdf [file jp2c07983_si_001.pdf]

How Do Liquid-Junction Potentials and Medium Polarity at Electrode Surfaces Affect Electrochemical Analyses for Charge-Transfer Systems? (Supporting Information)

Maximillian F. Mayther,[a,=] Omar O'Mari,[b,=] Paul Flacke,[b] Dev Bhatt,[b] Samantha Andrews,[b] and Valentine I. Vullev*,[a,b,c,d]

[a] Department of Chemistry, University of California, Riverside, CA 92521, USA.
[b] Department of Bioengineering, University of California, Riverside, CA 92521, USA.
[c] Department of Biochemistry, University of California, Riverside, CA 92521, USA.
[d] Materials Science and Engineering Program, University of California, Riverside, CA 92521, USA.

[=] Equal contribution
* vullev@ucr.edu

Table of Contents

Materials………………………………………………………….. S2

Analytical Electrochemistry……………………………………… S2

Global-Fit Analysis……………………………………………... S6

Optical Spectroscopy……………………………………………...S7

Refractometry…………………………………………………….. S7

Computational Analysis………………………………………….. S8

Supporting-Information References………………………………… S12

Materials

The samples 9-nitroanthracene (NA), 1-nitropyrene (NP) and coumarin 153 (C153) were purchased from Sigma-Aldrich; 10-methylphenothiazine (Ptz), 1-naphthylamine (AN) and 1-aminopyrene (AP) were obtained from Alfa Aesar; and 1-nitronaphthalene (NN) – from Spectrum Labs. The electrochemistry-grade supporting electrolyte, tetrabutylammonium hexafluorophosphate ($(n\text{-}C_4H_9)_4NPF_6$), was acquired from Sigma-Aldrich. The anhydrous solvents for the electrochemical measurements, dichloromethane (CH_2Cl_2) and acetonitrile (CH_3CN) were obtained from Acros Organics whilst benzonitrile (C_6H_5CN) was purchased from Sigma-Aldrich. The spectroscopy-grade solvents for the optical measurements, dichloromethane (CH_2Cl_2) and acetonitrile (CH_3CN) were attained from Thermo Fisher Scientific, whereas the solvent benzonitrile (C_6H_5CN) was obtained from Sigma-Aldrich.

Analytical Electrochemistry

Cyclic voltammetry is conducted using Reference 600™ Potentiostat/Galvanostat/ZRA (Gamry Instruments, PA, U.S.A.), connected to a three-electrode cell, as previously described.[1,2] Anhydrous aprotic solvents with different polarity, dichloromethane (CH_2Cl_2), benzonitrile (C_6H_5CN), and acetonitrile (CH_3CN) are employed with different concentrations of $(n\text{-}C_4H_9)_4NPF_6$ as a supporting electrolyte. Prior to recording each voltammogram, the sample is extensively purged with argon while maintaining its volume constant by adding more of the anhydrous solvent. For each solvent, a set of voltammograms is recorded where the electrolyte concentration is increased from 25 mM to 200 mM in increments of 25 mM.

The half-wave potentials, $E^{(1/2)}$, are determined form the midpoints between the cathodic and anodic peak potentials for reversible and quasi-reversible voltammograms; and from the inflection points of the anodic and cathodic waves for irreversible oxidation and reduction, respectively. The anodic and cathodic peak potentials, E_a and E_c, respectively, are determined from the zero points of the first derivatives of the voltammograms, i.e., the potentials where $\partial I/\partial E = 0$ at $\partial E/\partial t =$ constant. The inflection points are determined from the zero point of the second derivatives of the voltammograms, $\partial^2 I/\partial E^2 = 0$ at $\partial E/\partial t =$ constant.[2] The second derivatives of reversible and quasi-reversible voltammograms show that the inflection-point potentials are quite close to the mid-points between E_a and E_c, ensuring the reliability for the estimates of $E^{(1/2)}$ from the inflection points of irreversible voltammograms. The voltammograms are recorded at a scan rate of 100 and 150 mV s^{-1}. To correct for potential drifts in the reference electrode (which is SCE, connected with the cell via a salt bridge), ferrocene is used as a standard ($E^{(1/2)} = 0.45 \pm 0.01$ V vs. SCE for CH_3CN with 100 mM $(n\text{-}C_4H_9)_4NBF_4$).[3] Voltammograms of the ferrocene standard are recorded before and after each set of measurements.[4,5]

The voltammograms of Ptz in CH_2Cl_2 and C_6H_5CN show single anodic and cathodic waves consistent with single-electron reversible oxidation as reported for phenothiazine derivatives (Figure S1). For CH_3CN, however, concentrated Ptz samples or when the surface of the working electrode is not cleaned after each scan, we observe a shoulder on the anodic wave (Figure S1a). Keeping the Ptz concentration below about 5 mM suppresses this behavior. Overall, an increase in sample concentrations induces positive shift in the anodic peak that is larger than the negative shift of the cathodic one. An increase in the cell resistance, R (i.e., for low electrolyte concentrations and low solvent polarity), enhances these shifts. Therefore, lowering in the Faradaic current, by decreasing sample concentration, decreases the separation between the anodic and cathodic peak potentials, improves the appearance of the voltammograms, and induces a negative shift in $E^{(1/2)}$. Such concentration-induced shifts in $E^{(1/2)}$ can exceed 100 mV (Figure S1b). For this study, therefore, we implement sample concentrations of about 3 mM, minimizing the distortion of the voltammograms for low C_{el}.

The voltage drop, originating from the series cell resistance, R, further distorts the appearance of the voltammograms specially for non-polar solvents and low electrolyte concertation. The magnitudes of the current, i, at the anodic and the cathodic peak maxima are not the same, which warrants shifts in $E^{(1/2)}$ due to the voltage drop, iR. To correct for this effect we carry out cyclic voltammetry measurements with positive feedback iR

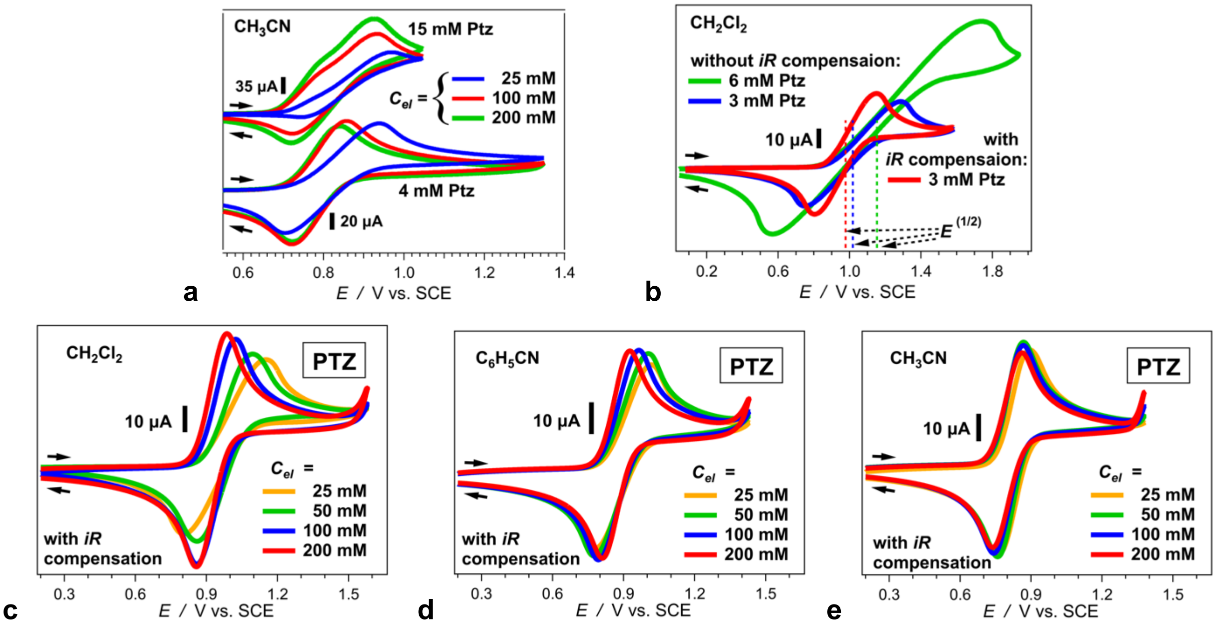

Figure S1. Electrochemical oxidation of Ptz. (a) Cyclic voltammograms of 4 mM and 15 mM Ptz in CH_3CN with different C_{el}. without implementation of iR compensation (v = 100 mV s^{-1}). (b) Cyclic voltammograms of 3 mM and 6 mM Ptz in CH_2Cl_2 with different C_{el}. (v = 100 mV s^{-1}). The two voltammograms for 3 mM Ptz samples show the changes upon implementation of iR compensation. The vertical dashed lines indicate the half-wave reduction potentials determined for each of the voltammograms. (c-e) Cyclic voltammograms of Ptz in CH_2Cl_2, C_6H_5CN and CH_3CN with iR compensation implemented for different concentrations of electrolyte, C_{el} (v = 150 mV s^{-1}).

compensation as implemented by Reference 600™ potentiostat. For the iR compensation, we resort to electrochemical impedance spectroscopy (ESI) to obtain estimates for the resistance, R_u, between the working electrode and the bridge with the reference electrode for the cell (Scheme 1) filled with the electrolyte solutions without any Ptz or NA analyte (Table S1). For each sample, we introduce the R_u values corresponding to the solvent and the electrolyte concentration it comprises, and run the sweeps with the positive feedback iR compensation on, which dynamically adjusts the recorded voltage for the iR_u as the cell current changes, yielding corrected values corresponding to the potential at the surface of the working electrode.

Table S1. Cell resistances R_u / Ω estimated from ESI measurements using Reference 600™ Potentiostat/Galvanostat/ZRA (Gamry Instruments, PA, U.S.A.) for the different solvents and C_{el} concentrations using $(n\text{-}C_4H_9)_4NPF_6$ as a supporting electrolyte.

solvent	C_{el} / mM							
	25	50	75	100	125	150	175	200
CH_2Cl_2	4280	2220	1390	1280	1110	1090	850	680
C_6H_5CN	1630	869	793	707	702	640	580	575
CH_3CN	1090	619	462	398	311	299	284	256

The iR compensation improves immensely the appearance of the cyclic voltammograms by decreasing the separation between the anodic and the cathodic peak potentials. Nevertheless, the difference between the $E^{(1/2)}$ extracted from voltammograms (for 3 mM Ptz samples) with and without iR compensation do no exceed 50 mV (Figure S2). This finding concurs with the fact that iR compensation has a stronger impact on the kinetic than on the thermodynamic electrochemical analysis. For example, voltammograms without implemented iR compensation can yield huge errors in estimating the interfacial charge-transfer rate constants. Conversely, estimates of reduction potentials should be practically invariant to the iR of the cell. Even though the iR-induced

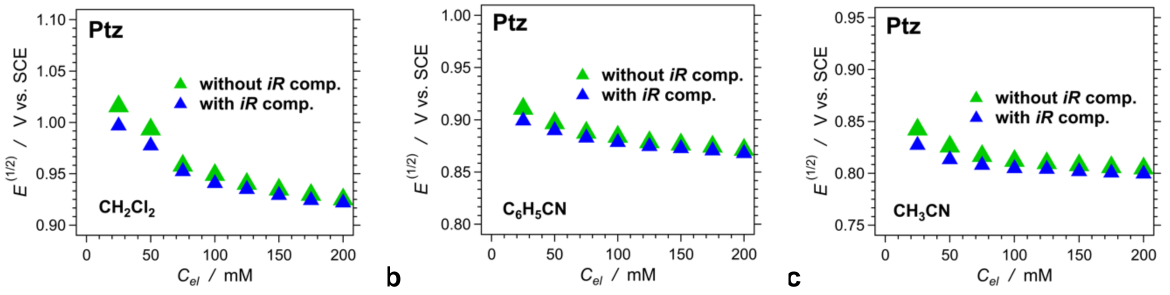

Figure S2. Effect of iR compensation on the half-wave reduction potentials of $Ptz^{\bullet+}$ for varied electrolyte concentration, C_{el}, in (a,) dichloromethane, (b) benzonitrile, and (c) acetonitrile.

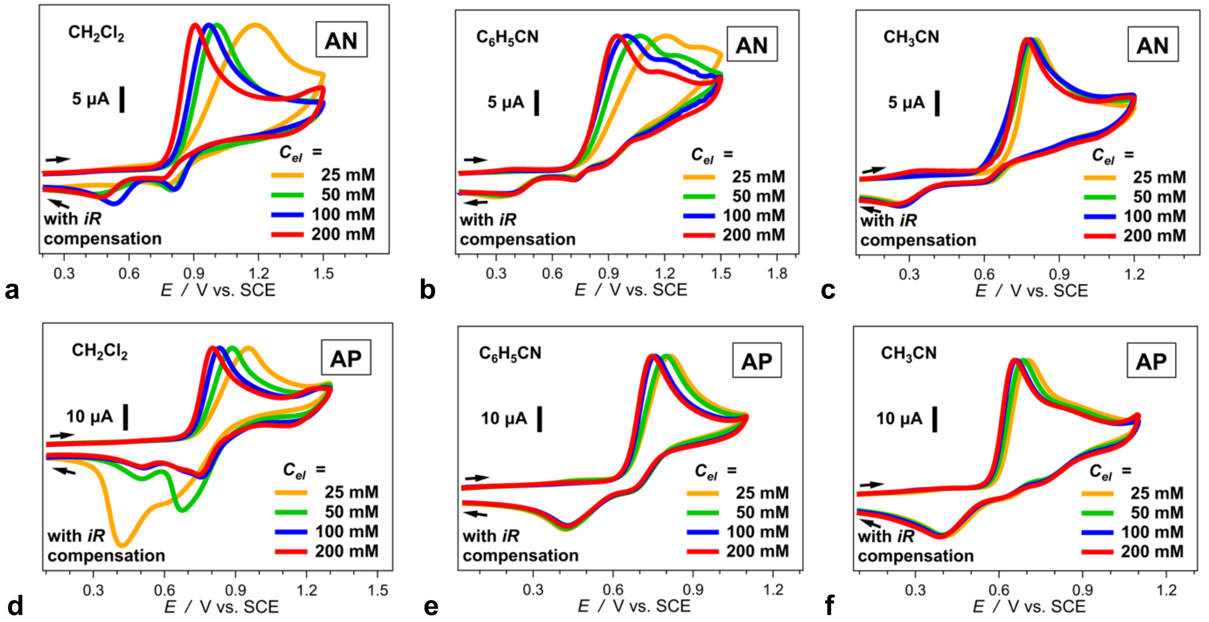

Figure S3. Cyclic voltammograms of (a-c) AN and (d-f) AP, compensated for the iR the potential drops, showing irreversible oxidation in (a,d) dichloromethane, (b,e) benzonitrile, and (c,f) acetonitrile in the presence of different concentrations of supporting electrolyte, C_{el} (v = 150 mV s^{-1}).

changes in $E^{(1/2)}$ do not exceed $2k_BTF^{-1}$, the reduction potentials of $AN^{\bullet+}$, $Ptz^{\bullet+}$, $AP^{\bullet+}$, NN, NA and NP used in this study are from voltammograms with implemented iR compensation (Figure S3 and S4).

For voltammogram originating from a single oxidation step of a single species, the potential at the inflection point, $E^{(i)}$, of the anodic wave has close values to $E^{(1/2)}$ obtained from the average between the anodic and cathodic peak potentials. For Ptz in the different solvents with different C_{el}, $E^{(i)}$ and $E^{(1/2)}$ are quite similar (Figure S5). This finding further validates the use of the $E^{(i)}$ values for estimates of $E^{(1/2)}$ for the samples that manifest irreversible oxidation or reduction behavior. For oxidation, the values of $E^{(i)}$ are extracted from the anodic waves (Figure S3), and for reduction – from the cathodic waves (Figure S4d-f).

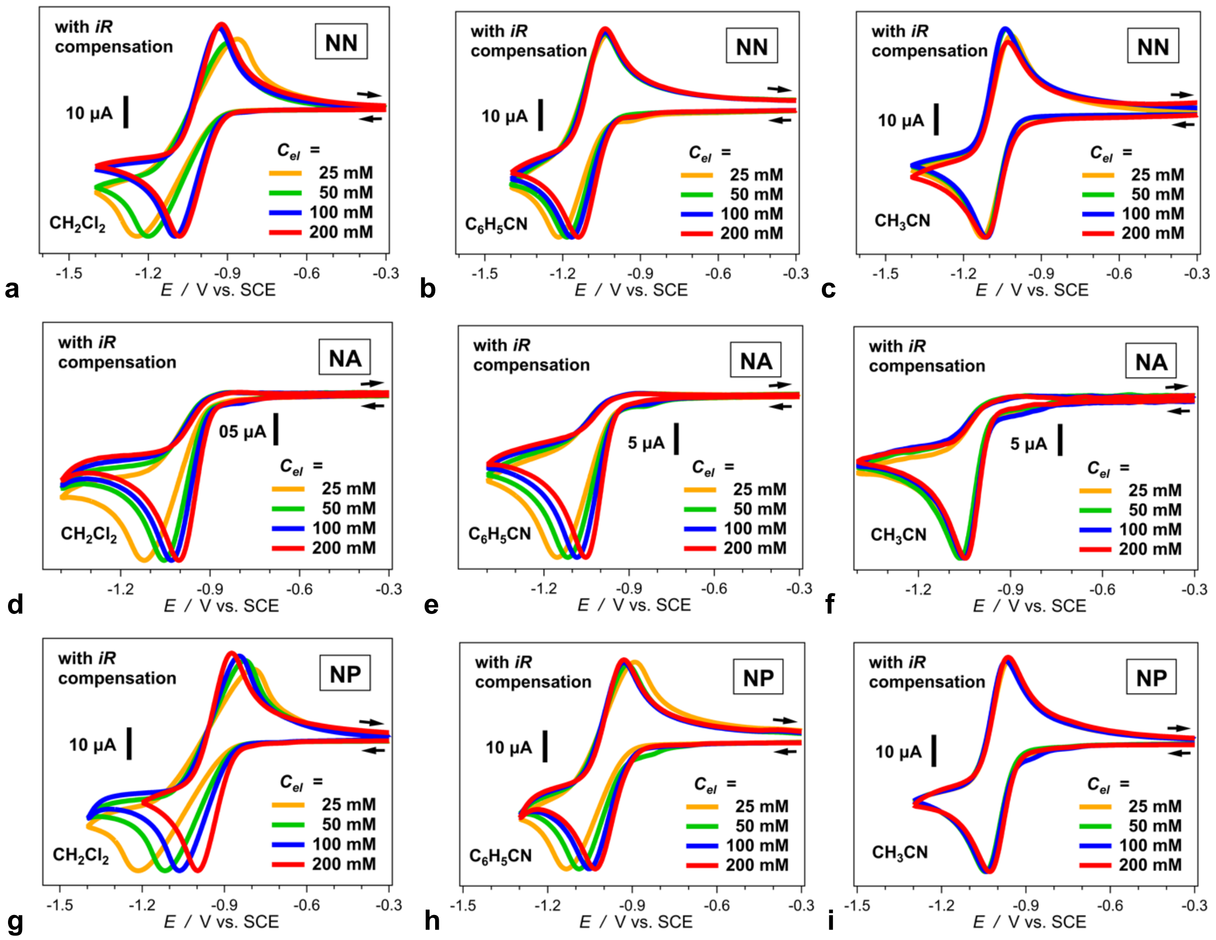

Figure S4. Cyclic voltammograms of (a-c) NN, (d-f) NA,[6,7] and (g-i) NP, compensated for the *iR* the potential drops, showing reversible reduction of NN and NP and irreversible reduction of NA for (a,d,g) dichloromethane, (b,e,h) benzonitrile, and (c,f,i) acetonitrile in the presence of different concentrations of supporting electrolyte, C_{el} (ν = 150 mV s^{-1}).

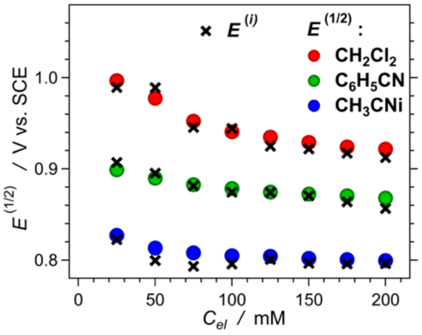

Figure S5. Comparison between the half-wave reduction potentials, $E^{(1/2)}$, of Ptz obtained from the average between the anodic and cathodic peak potentials, with the inflection-point potentials, $E^{(i)}$, obtained from the first inflection points of the anodic waves. The values of $E^{(1/2)}$ and $E^{(i)}$ are obtained from voltammograms with implemented *iR* compensation.

Global-Fit Analysis

For the global-fit analysis of the dependence of the reduction potentials, $E^{(1/2)}$, on the estimated polarity at the electrode surface, $f_B^{(E)}$ (Figure 4), we employ the Global Fit package as implemented by Igor Pro, v. 8.02 (Wavemetrics, Portland, OR, USA). Using eq. 5, we calculate ($f_B^{(E)} - f^{(\mathrm{E})}_{B,\,C_{el}=0}$) for each donor-acceptor pair in the different solvents with the different concentrations of electrolyte. For these initial estimates of ($f_B^{(E)} - f^{(\mathrm{E})}_{B,\,C_{el}=0}$), $S_{1/r}^{(\mathrm{DFT})}$ is calculated using radii obtained from DFT calculations and input in eq. 5. During the fitting, ($f_B^{(E)} - f^{(\mathrm{E})}_{B,\,C_{el}=0}$) is continuously adjusted by multiplying it by $S_{1/r} / S_{1/r}^{(\mathrm{DFT})}$, where $S_{1/r}$ is one of the fitting parameters.

The reduction potentials of each of the six species in each of the three solvents show almost linear dependence on ($f_B^{(E)} - f^{(\mathrm{E})}_{B,\,C_{el}=0}$) (Figure S6). These 18 sets of data were fit simultaneously using the flowing function: $E^{(1/2)}(f_B^{(\mathrm{E})} - f^{(\mathrm{E})}_{B,\,C_{el}=0}) = E^{(1/2)}(f_B^{(\mathrm{E})} = 0) + S\,(f_B^{(E)} - f^{(\mathrm{E})}_{B,\,C_{el}=0}) + E_{\mathrm{LJ}}^{(s)} + E_{\mathrm{LJ}}^{(el)}$, where the intercept $E^{(1/2)}(f_B^{(\mathrm{E})} = 0)$ represents the reduction potential in vacuum, i.e., for $\varepsilon = 1$, and the slope is $S = 0.5\, S_{1/r}\, (8\,\pi\, F\, \varepsilon_0)^{-1}$. As a fitting parameter, $f^{(\mathrm{E})}_{B,\,C_{el}=0}$ was allowed to optimize so that: (1) the six slopes, S, of each donor-acceptor pair match for all three solvents ($S_{donor} = -S_{acceptor}$); (2) the intercepts for each species in all three solvents are the same; (3) $E_{\mathrm{LJ}}^{(s)}$ for each solvent is the same (indeed, for the acetonitrile samples, $E_{\mathrm{LJ}}^{(s)}$ is kept at zero), and (4) $E_{\mathrm{LJ}}^{(s)}$ shows identical dependence on C_{el} for all 18 sets of data. Adding the values of $f^{(\mathrm{E})}_{B,\,C_{el}=0}$, obtained from the global fit, to ($f_B^{(E)} - f^{(\mathrm{E})}_{B,\,C_{el}=0}$) allows estimating the polarity $f_B^{(E)}$ that the six species experience at the surface of the working electrode in the different solvents comprising different concentrations of electrolyte (Figure 4, 8).

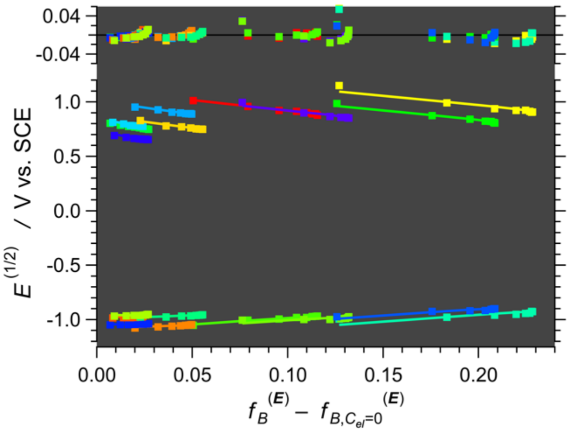

Figure S6. Global fits (solid lines) of the reduction potentials of $AN^{\bullet+}$, $Ptz^{\bullet+}$, $AP^{\bullet+}$, NN, NA and NP for dichloromethane, benzonitrile and acetonitrile vs. the relative polarity at the surface of the working electrode, estimated using eq. 5. The dots represent the data points, along with the residuals from the fits (on the top of the graph).

Optical Spectroscopy

Steady-state absorption spectra are recorded in a transmission mode using a JASCO V-670 spectrophotometer (Tokyo, Japan).[8-13] The steady-state emission spectra are measured, using a FluoroLog-3 spectrofluorometer (Horiba-Jobin-Yvon, Edison, NJ, USA) as previously reported.[14-16] The optical spectra are recorded in wavelength scales, i.e., absorbance and emission intensity vs. wavelength in nanometers (λ / nm). To convert them to energy scales, i.e., $\mathcal{E}$, it is important not only to convert the abscissa, i.e., $\mathcal{E} = h\,c\,\lambda^{-1}$, but also to divide the absorbance by $\mathcal{E}$ and the emission intensity by $\mathcal{E}^5$, i.e., $A(\mathcal{E}) = A(\lambda)\ \mathcal{E}^{-1}$ and $F(\mathcal{E}) = F(\lambda)\ \mathcal{E}^{-5}$. The optical spectra, plotted against $\mathcal{E}$, were fit to sums of Gaussian functions. The positions of the largest-amplitude components were ascribed to the spectral maxima (Figure 5b), which provide estimates for the Stokes' shifts, $\Delta\mathcal{E}$ (Figure 6b).

The linear correlation between the Stokes' shifts and $f_O(\varepsilon, n^2)$ for the neat solvents yields the slope and the intercept required for estimating the polarities of the electrolyte solutions, eq. 6a (Figure S5a). Plugging these results back into eq. 6a, along with the Stokes' shifts for the different electrolyte solitons (Figure 6b) yields the values of $f_O(\varepsilon, n^2)$ for different C_{el} in the three different solvents (Figure S5b). For the validity of the LMO analysis, $|\Delta\boldsymbol{\mu}|$ should be invariant to the medium polarity. Computational analysis shows about a 10% decrease in $|\Delta\boldsymbol{\mu}|$ when varying the solvent from CH_3CN to CH_2Cl_2, which is consistent with the amplification of the Onsager reaction field in the excited state that is more polar that S_0. Despite this 10% fluctuation, within the polarity range of these three solvents, the LMO analysis can still produce meaningful trends for the media polarity, and especially for the effects of C_{el} on the polarity that solvated molecular species experience.

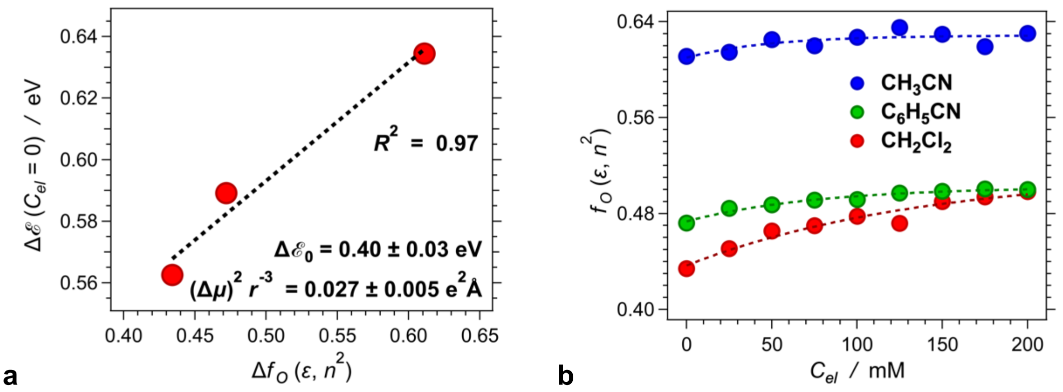

Figure S5. LMO analysis of the C153 Stokes' shifts for different solvents and C_{el}. (a) Linear fit of $\Delta\mathcal{E}$ vs. $f_O(\varepsilon, n^2)$ for the neat solvents, i.e., $C_{el} = 0$. The slope suggests for $|\Delta\boldsymbol{\mu}|$ of about 4 D for $r \approx 3$ Å. (b) Dependence of the Onsager polarity, $f_O(\varepsilon, n^2)$, of the electrolyte solutions on their concentration. Introducing the Stokes' shifts of C153 dissolved in the electrolyte solutions, along with the slope and intercept from Figure S5a, to the LMO equation, eq. 6a, allows solving for $f_O(\varepsilon, n^2)$.

Refractometry

The refractive indices (n) of the electrolyte solutions and the solvents are measured at an ambient temperature and pressure using Rudolph Research J357 Automatic Refractometer (Rudolph Research Analytical, Hackettstown, NJ) at the wavelength of the D-line of sodium, 589.3 nm, with an estimated ±0.00002 precision. The refractometer operates on an artificial sapphire prism with automatic temperature control. The liquid samples solutions are allowed to reach thermal equilibrium prior to recording their refractive indices.

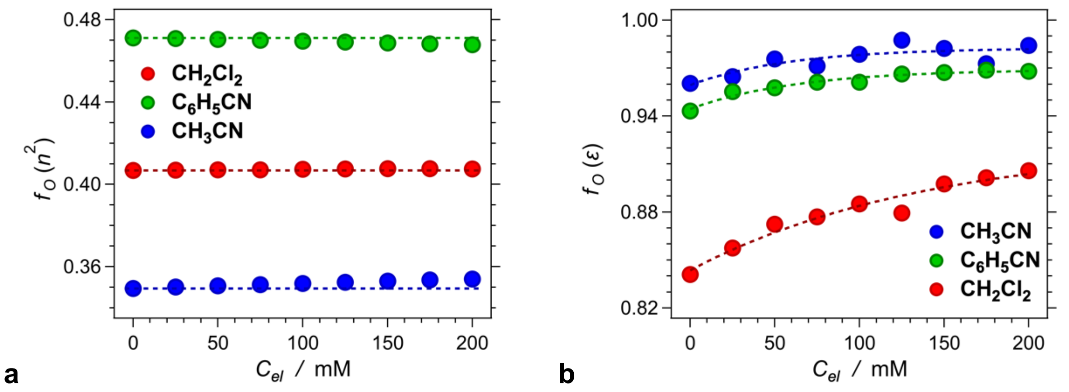

Figure S7. C_{el} dependence of the Onsager polarities originating from (a) the dynamic, n^2, and (b) the static, ε, dielectric properties of the solvating media, obtained from refractometry, i.e., n, and LMO analysis, i.e., ε.

.

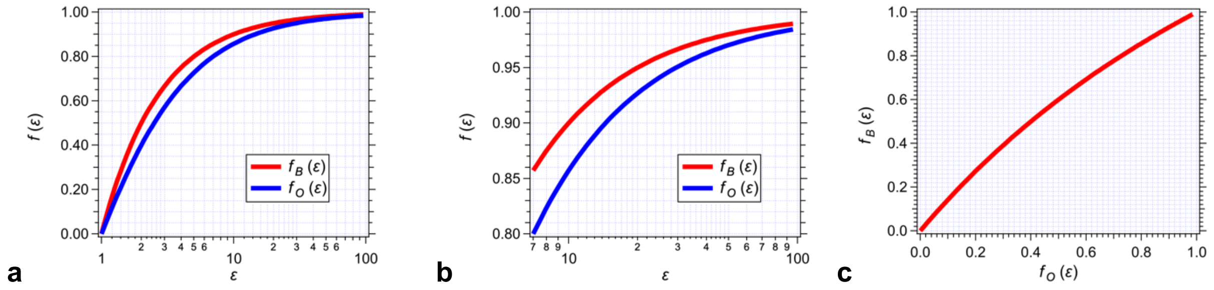

Figure S8. Relationship between the Born polarity, $f_B(\varepsilon)$, and the Onsager polarity, $f_O(\varepsilon)$, originating from the static dielectric properties of the media. (a) Dependence of the Born and Onsager polarities on the static dielectric constant. (b) The same dependence of $f_B(\varepsilon)$ and $f_O(\varepsilon)$ on ε for polarities most relevant for electrochemical analysis, i.e., for $7 \lesssim \varepsilon \lesssim 100$. (c) Graphical representation of the relationship between $f_B(\varepsilon)$ and $f_O(\varepsilon)$, eq. 7. For liquids, the static dielectric constant of the media does not drop below about 2. Thus, $f_B \lesssim 0.5$ is irrelevant for condensed phase liquid analytes. On the other side of the spectrum, increasing the medium static dielectric constant makes $f_B(\varepsilon)$ and $f_O(\varepsilon)$ asymptotically approach unity. At $\varepsilon = 100$, $f_B(\varepsilon)$ is already around 0.955. Any further increase in ε will not gain significant benefits regarding stabilization of charged analyte by the solvating medium, unless certain specific solvent-solute interactions are important.

The measured refractive indices are used for calculating $f_O(n^2)$ for the electrolyte solutions, eq. 6c (Figure S7a). Adding the obtained $f_O(n^2)$ values to $f_O(\varepsilon, n^2)$ (Figure S5b) yields $f_O(\varepsilon)$, eq. 6b (Figure S7b). Using eq. 7a, allows estimating the Born polarities for the bulk solutions, $f_B^{(S)}$, from $f_O(\varepsilon)$ (Figure 3b). Strictly speaking, for polar solvents, f_B and $f_O(\varepsilon)$ have a similar dependence on the static dielectric constant, ε (Figure S8). Therefore, this conversion from $f_O(\varepsilon)$ to f_B is important for solutions with medium to low polarity, i.e., for $2 \lesssim \varepsilon \lesssim 15$.

Computational Analysis

Molecular structures of the redox species, Ptz, NA, ANA and Tha, and their radical ions are optimized using the Gaussian 09 program package.[17] B3LYP functional[18,19] with in the density-functional theory (DFT) framework along with the Grimme's[20] dispersion DFT-D3 correction employing the 6-311+G(d,p) basis set. For the radical-ion calculations, spin-unrestricted DFT calculations are utilized. Solvation effects are studied using the integral equation formalism variant of the polarizable continuum model (IEFPCM).[21] Solvents has minimal effect on the spin density distribution (SDD) of the radical ions of these structures (Figure S9-S12).

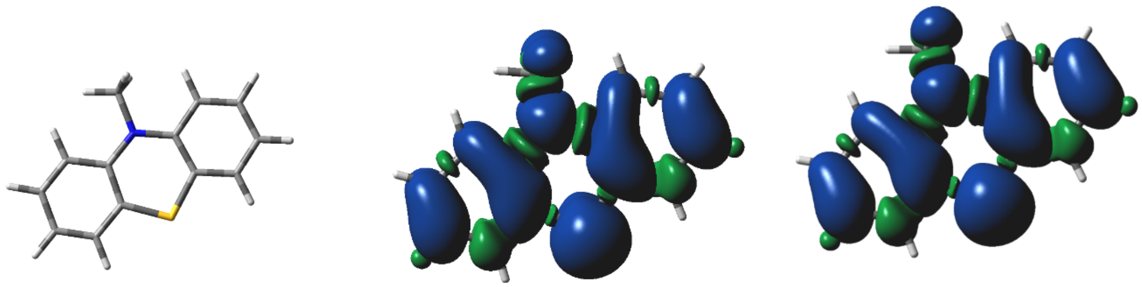

$Ptz^{•+}$ CH_2Cl_2 CH_3CN

Figure S9. Molecular structure $Ptz^{•+}$ in dichloromethane (CH_2Cl_2) and acetonitrile (CH_3CN) and their spin-density distribution, SDD (for CH_2Cl_2 and CH_3CN), of the singly oxidized 10-methylphenothiazine, obtained from DFT calculations (blue – excess spin up; green – excess spin down).

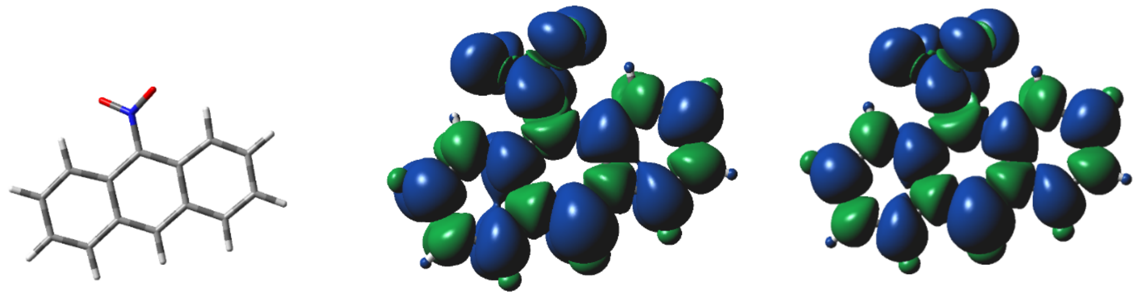

$NA^{•-}$ CH_2Cl_2 CH_3CN

Figure S10. Molecular structure $NA^{•-}$ in dichloromethane (CH_2Cl_2) and acetonitrile (CH_3CN) and their spin-density distribution, SDD (for CH_2Cl_2 and CH_3CN), of the singly reduced 9-nitroanthracene, obtained from DFT calculations (blue – excess spin up; green – excess spin down).

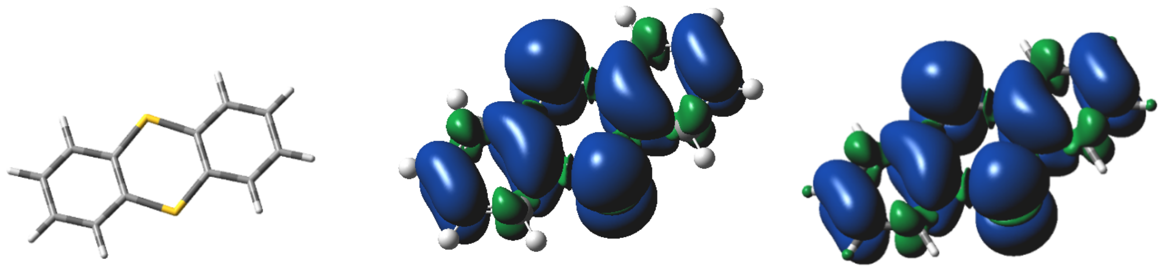

$Tha^{•+}$ CH_2Cl_2 CH_3CN

Figure S11. Molecular structure $Tha^{•+}$ in dichloromethane (CH_2Cl_2) and acetonitrile (CH_3CN) and their spin-density distribution, SDD (for CH_2Cl_2 and CH_3CN), of the singly oxidized Thianthrene, obtained from DFT calculations (blue – excess spin up; green – excess spin down).

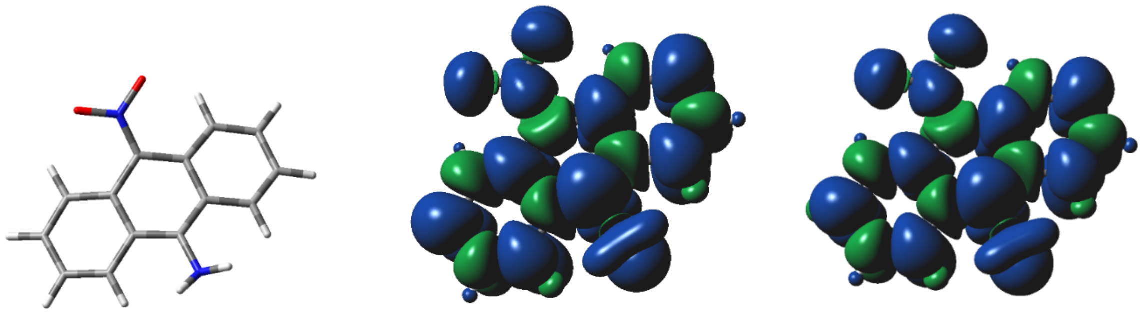

ANA•⁻ **CH_2Cl_2** **CH_3CN**

Figure S12. Molecular structure ANA•⁻ in dichloromethane (CH_2Cl_2) and acetonitrile (CH_3CN) and their spin-density distribution, SDD (for CH_2Cl_2 and CH_3CN), of the singly reduced 9-amino-10-nitroanthracene, obtained from DFT calculations (blue – excess spin up; green – excess spin down).

The molecular structures of coumarin 153, C153 (Figure S13), are optimized using the Gaussian 09 program package[17] within the Hartree-Fock (HF) theory framework,[22] employing the 6-311++G(2dF,3p) basis set. For the excited-states' calculations of C153, we resort to the configuration interaction singles (CIS) method[23] using single-excitation CI, i.e., CI-Singles. Solvation effects are implemented with the integral equation formalism variant of the polarizable continuum model (IEFPCM).[21]

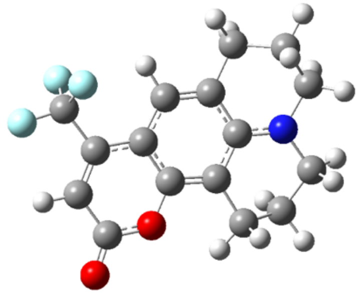

Figure S13. Structure of C153.

From the optimized structures, we estimate the permanent electric dipole moments of the ground and the fluorescent excited state. The dipoles of the ground and the excited state are practically parallel, and the solvents have negligible effects on their magnitudes and orientation (Table S2).

Table S2. Permanent electric dipole moments of the ground and the excited state of C153, i.e., $\boldsymbol{\mu}_0$ and $\boldsymbol{\mu}^*$ for different solvents.

solvent	$\lvert\boldsymbol{\mu}_0\rvert$ / D [a]	$\lvert\boldsymbol{\mu}^*\rvert$ / D [a]	α / deg [b]	$\Delta\mu$ / D [c]	$\lvert\Delta\boldsymbol{\mu}\rvert$ / D [d]
**CH_2Cl_2**	9.32	12.39	1.29	3.07	3.08
**C_6H_5CN**	9.63	13.01	1.33	3.38	3.39
**CH_3CN**	9.67	13.12	1.40	3.45	3.46

[a] Magnitudes of the ground and excited-state dipoles, i.e., $\mu = \lvert\boldsymbol{\mu}\rvert$. [b] Angle between $\boldsymbol{\mu}_0$ and $\boldsymbol{\mu}^*$. [c] Difference between the magnitudes of $\boldsymbol{\mu}_0$ and $\boldsymbol{\mu}^*$, i.e., $\Delta\mu = \lvert\boldsymbol{\mu}^*\rvert - \lvert\boldsymbol{\mu}_0\rvert$. [d] Magnitudes of the vector differences between $\boldsymbol{\mu}_0$ and $\boldsymbol{\mu}^*$, i.e., $\lvert\Delta\boldsymbol{\mu}\rvert = \lvert\boldsymbol{\mu}^* - \boldsymbol{\mu}_0\rvert$, which is equivalent to $\lvert\Delta\boldsymbol{\mu}\rvert^2 = \lvert\boldsymbol{\mu}^*\rvert^2 + \lvert\boldsymbol{\mu}_0\rvert^2 - 2\,\lvert\boldsymbol{\mu}^*\rvert\,\lvert\boldsymbol{\mu}_0\rvert \cos\alpha$. $\boldsymbol{\mu}_0$ and $\boldsymbol{\mu}^*$ are practically colinear, with an angle between them less than 1.5°, $\lvert\Delta\boldsymbol{\mu}\rvert \approx \Delta\mu$.

The natural transition orbitals (NTOs) [24] represents the electronic transition of optical excitation and emission, with occupation numbers of 0.97 or higher. Varying the solvent has minimal impacts on the NTOs (Figure 5, S14, S15).

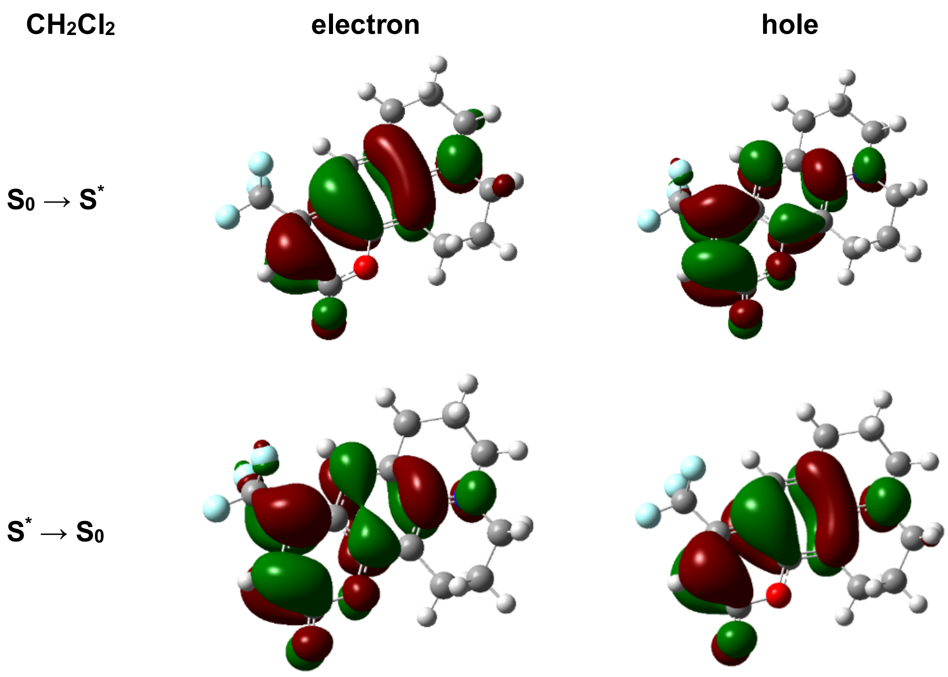

Figure S14. Dominant NTOs for the optical $S_0 \rightarrow S^*$ and $S^* \rightarrow S_0$ transitions of C153 with CH_2Cl_2 introduced as a solvating medium.

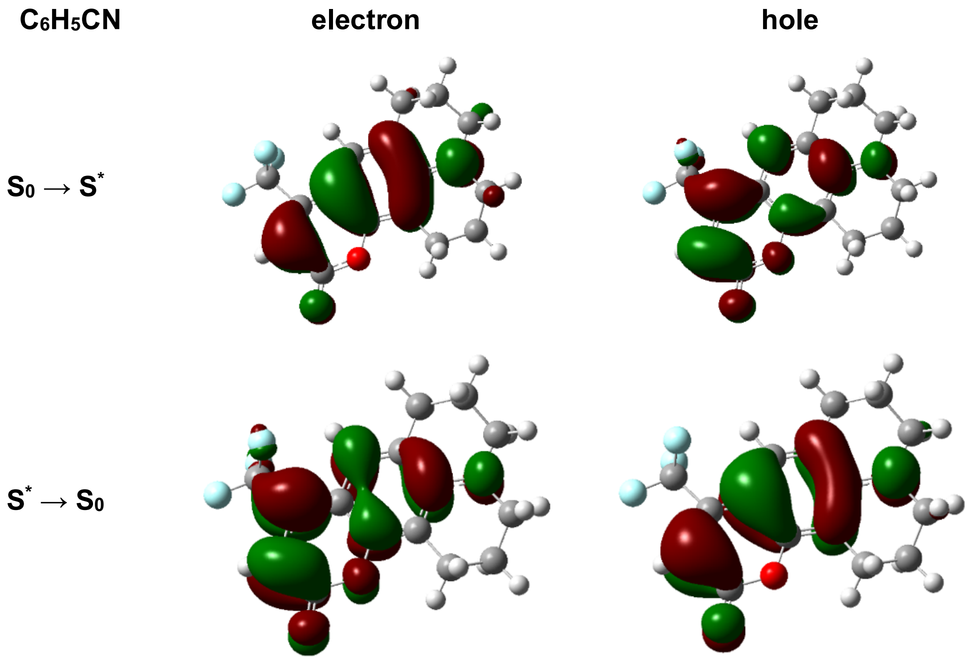

Figure S15. Dominant NTOs for the optical $S_0 \rightarrow S^*$ and $S^* \rightarrow S_0$ transitions of C153 with C_6H_5CN introduced as a solvating medium.

References:

(1) Purc, A. Espinoza; E. M.; Nazir, R.; Romero, J. J.; Skonieczny, K.; Jeżewski, A.; Larsen, J. M.; Gryko, D. T.; Vullev, V. I. Gating That Suppresses Charge Recombination–The Role of Mono-N-Arylated Diketopyrrolopyrrole. *J. Am. Chem. Soc.* **2016,** 138, 12826–12832.

(2) Espinoza, E. M.; Larsen, J. M.; Vullev, V. I. What Makes Oxidized N-Acylanthranilamides Stable? *J. Phys. Chem. Lett.* **2016**, 7, 758–764.

(3) Bao, D.; Millare, B.; Xia, W.; Steyer, B. G.; Gerasimenko, A. A.; Ferreira, A.; Contreras, A.; Vullev, V. I. Electrochemical Oxidation of Ferrocene: A Strong Dependence on the Concentration of the Supporting Electrolyte for Nonpolar Solvents. *J. Phys. Chem. A* **2009,** 113, 1259–1267.

(4) Bao, D.; Ramu, S.; Contreras, A.; Upadhyayula, S.; Vasquez, J. M.; Beran, G.; Vullev, V. I. Electrochemical Reduction of Quinones: Interfacing Experiment and Theory for Defining Effective Radii of Redox Moieties. *J. Phys. Chem. B* **2010**, 114, 14467–14479.

(5) Espinoza, E. M.; Xia, B.; Darabedian, N.; Larsen, J. M.; Nuñez, V.; Bao, D.; Mac, J. T.; Botero, F.; Wurch, M.; Zhou, F.; Vullev, V. I. Nitropyrene Photoprobes: Making Them, and What Are They Good for? *Eur. J. Org. Chem.* **2016**, 343–356.

(6) Kitagawa, T.; Ichimura, A. Polarographic Behavior of 9-Nitroanthracene and 9,10-Dinitroanthracene in Dimethylformamide. *Bull. Chem. Soc. Jap.* **1973**, 46, 3792–3795.

(7) Mendkovich, A. S.; Churilina, A. P.; Mikhal'chenko, L. V.; Gul'tyai, V. P. Effect of the structure of the aromatic system on the rate of dimerization of radical anions of aromatic nitro compounds. *Russ. Chem. Bull.* **1990**, *39*, 1348-1351.

(8) Bahmani, B.; Gupta, S.; Upadhyayula, S.; Vullev, V. I.; Anvari, B. Effect of polyethylene glycol coatings on uptake of indocyanine green loaded nanocapsules by human spleen macrophages in vitro. *J. Biomed. Opt.* **2011**, *16*, 051303.

(9) Bahmani, B.; Lytle, C. Y.; Walker, A. M.; Gupta, S.; Vullev, V. I.; Anvari. B. Effects of nanoencapsulation and PEGylation on biodistribution of indocyanine green in healthy mice: quantitative fluorescence imaging and analysis of organs. *Int. J. Nanomedicine* **2013,** 1609-1620.

(10) Nuñez, V.; Upadhyayula, S.; Millare, B.; Larsen, J. M.; Hadian, A.; Shin, S.; Vandrangi, P.; Gupta, S.; Xu, H.; Lin, A. P.; Georgiev, G. Y.; Vullev, V. I. Microfluidic Space-Domain Time-Resolved Emission Spectroscopy of Terbium(III) and Europium(III) Chelates with Pyridine-2,6-Dicarboxylate. , *Anal. Chem.* **2013**, 85, 4567–4577.

(11) Bahmani, B.; Guerrero, Y.; Bacon, D.; Kundra, V.; Vullev, V. I.; Anvari, B. Functionalized polymeric nanoparticles loaded with indocyanine green as theranostic materials for targeted molecular near infrared fluorescence imaging and photothermal destruction of ovarian cancer cells. *Laser Surg. Med.* **2014**, 46, 582–592.

(12) Chau, K.; Millare, B.; Lin, A.; Upadhyayula, S.; Nuñez, V.; Xu, H.; Vullev, V. I. Dependence of the quality of adhesion between poly(dimethylsiloxane) and glass surfaces on the composition of the oxidizing plasma. *Microfluid. Nanofluid.* **2011**, 10, 907–917.

(13) Gupta, S.; Chatni, M. R.; Rao, A. L. N.; Vullev, V. I.; Wang, L. V.; Anvari, B. Virus-mimicking nano-constructs as a contrast agent for near infrared photoacoustic imaging. *Nanoscale* **2013**, 5, 1772-1776.

(14) Wan, J.; Ferreira, A.; Xia, W.; Chow, C. H.; Takechi, K.; Kamat, P. V.; Jones, G.; Vullev, V. I. Solvent dependence of the charge-transfer properties of a quaterthiophene–anthraquinone dyad. , *J. Photochem. Photobiol. A* **2008**, *197*, 364-374.

(15) Lu, H., Bao, D., Penchev, M., Ghazinejad, M., Vullev, V. I., Ozkan, C. S., & Ozkan, M. Pyridine-Coated Lead Sulfide Quantum Dots for Polymer Hybrid Photovoltaic Devices. *Advanced Science Letters* **2010**, *3*, 101-109.

(16) Bongsu Jung; Vullev, V. I.; Anvari, B. Revisiting Indocyanine Green: Effects of Serum and Physiological Temperature on Absorption and Fluorescence Characteristics. *Quantum Electron.* **2014**, 20, 149–157.

(17) Frisch, M. J.; Trucks, G. W.; Schlegel, H. B.; Scuseria, G. E.; Robb, M. A.; Cheeseman, J. R.; Scalmani, G.; Barone, V.; Mennucci, B.; Petersson, G. A.; Nakatsuji, H. , in *Gaussian 09, Revision D.01,* Gaussian, Inc., Wallingford, **2013**.

(18) Becke, A. D. Density-functional thermochemistry. III. The role of exact exchange. *J. Chem. Phys.* **1993**, *98*, 5648-5652.
(19) Lee, C.; Yang, W.; Parr, R. G. Development of the Colle-Salvetti correlation-energy formula into a functional of the electron density. *Phys. Rev. B* **1988**, *37*, 785-789.
(20) Grimme, S.; Antony, J., Ehrlich; S., Krieg, H. A consistent and accurate ab initio parametrization of density functional dispersion correction (DFT-D) for the 94 elements H-Pu. *J. Chem. Phys*. **2010**, 132, 154104.
(21) Cossi, M.; Barone, V.; Cammi, R.; Tomasi, J. Ab initio study of solvated molecules: a new implementation of the polarizable continuum model. *Chem. Phys. Lett.* **1996**, *255*, 327-335.
(22) Roothaan, C. C. J. New Developments in Molecular Orbital Theory. *Rev. Mod. Phys.* **1951**, 23, 69-89.
(23) Foresman, J. B.; Head-Gordon, M.; Pople, J. A.; Frisch, M. J. Toward a systematic molecular orbital theory for excited states *J. Phys. Chem.*, **1992**, 96, 135-49.
(24) Martin, R. L. Natural transition orbitals. *Chem. Phys.* **2003**, 118, 4775-4777.
